# Variation in the benefits of multiple mating on female fertility in wild stalk‐eyed flies

**DOI:** 10.1002/ece3.3486

**Published:** 2017-10-24

**Authors:** Lara Meade, Elisabeth Harley, Alison Cotton, James M. Howie, Andrew Pomiankowski, Kevin Fowler

**Affiliations:** ^1^ Department of Genetics, Evolution and Environment University College London London UK; ^2^ CoMPLEX University College London London UK; ^3^ Bristol Zoological Society Bristol Zoo Gardens Clifton Bristol UK

**Keywords:** Diopsidae, ejaculate partitioning, mating systems, sperm depletion, wild‐caught flies

## Abstract

Polyandry, female mating with multiple males, is widespread across many taxa and almost ubiquitous in insects. This conflicts with the traditional idea that females are constrained by their comparatively large investment in each offspring, and so should only need to mate once or a few times. Females may need to mate multiply to gain sufficient sperm supplies to maintain their fertility, especially in species in which male promiscuity results in division of their ejaculate among many females. Here, we take a novel approach, utilizing wild‐caught individuals to explore how natural variation among females and males influences fertility gains for females. We studied this in the Malaysian stalk‐eyed fly species *Teleopsis dalmanni*. After an additional mating, females benefit from greatly increased fertility (proportion fertile eggs). Gains from multiple mating are not uniform across females; they are greatest when females have high fecundity or low fertility. Fertility gains also vary spatially, as we find an additional strong effect of the stream from which females were collected. Responses were unaffected by male mating history (males kept with females or in male‐only groups). Recent male mating may be of lesser importance because males in many species, including *T. dalmanni*, partition their ejaculate to maintain their fertility over many matings. This study highlights the importance of complementing laboratory studies with data on wild‐caught populations, where there is considerable heterogeneity between individuals. Future research should focus on environmental, demographic and genetic factors that are likely to significantly influence variation in individual female fecundity and fertility.

## 1 INTRODUCTION

Female mating with multiple males (polyandry) is found widely across many taxa (mammals: Clutton‐Brock, 1989; Ginsberg & Huck, 1989; birds: Griffith, Owens, & Thuman, 2002; fishes: Avise, Jones, Walker, & DeWoody, 2002; general: Jennions & Petrie, 2000; Zeh & Zeh, 2001) and is almost ubiquitous in insects (Arnqvist & Nilsson, 2000). While multiple mating is expected in males, as their reproductive success typically increases with the number of matings, it is less clearly beneficial for females. Female reproductive potential is thought to be realized after one or a few matings (Bateman, 1948), as females are assumed to be constrained by the greater investment they make in each of their offspring. This has led to an extensive literature considering potential benefits to females from multiple mating, in terms of increases to female survival, fecundity and fertility (Arnqvist & Nilsson, 2000; Hosken & Stockley, 2003; Yasui, 1998), and whether polyandry may be a mechanism to quell or mitigate intragenomic conflicts (Haig & Bergstrom, 1995; Zeh & Zeh, 1996). Additionally, females may gain indirect genetic benefits through increasing the genetic diversity or quality of offspring, but these are likely to be of secondary importance when females gain direct benefits from multiple mating (Slatyer, Mautz, Backwell, & Jennions, 2012; Yasui, 1998).

However rather less attention has been given to considering how variation among females impacts on the benefits of multiple mating. For instance, how does female condition, fecundity, or prior mating history alter the fitness consequences of further matings or polyandrous matings? Greater study in this area is needed in order to uncover the contexts in which multiple mating benefits, harms, or has no effect on females (House, Walling, Stamper, & Moore, 2009; Toft & Albo, 2015; Wright et al., 2013). In addition, there has been an over‐reliance on laboratory matings to investigate the consequence of multiple mating. While laboratory studies allow control and standardization (e.g., using virgins), assays may not fully reflect the natural history of mating experienced by females and males. Laboratory studies need to be complemented by experiments conducted on wild‐caught individuals, in situations that more closely replicate the natural range of conditions of female and male encounters.

Here, we apply these principles to consider the consequences of multiple mating on female fertility in the Malaysian stalk‐eyed fly *Teleopsis dalmanni*, when females vary in the degree of sperm limitation. In insects, it is widely found that sperm acquired in a single mating is insufficient to fertilize all of a female's eggs (Ridley, 1988; Wedell, Gage, & Parker, 2002). To maintain fertility, females may need to mate multiply to gain sufficient sperm supplies for egg laying throughout their adult life (Chevrier & Bressac, 2002; Fjerdingstad & Boomsma, 1998) or remate at regular intervals as sperm supplies dwindle (Drnevich, Papke, Rauser, & Rutowski, 2001; Fox, 1993; Wang & Davis, 2006). This implies that the fertility benefits of female remating will change with fluctuating environmental factors, such as the operational sex ratio, food availability, and the fertility of previous mates (Arnqvist & Nilsson, 2000; Cordero & Eberhard, 2003; Crean & Marshall, 2009; Fox, 1993; Navara, Anderson, & Edwards, 2012; Pitcher, Neff, Rodd, & Rowe, 2003; Rogers, Denniff, Chapman, Fowler, & Pomiankowski, 2008; Tuni, Albo, & Bilde, 2013). In line with this view, females may be able to modify their mating rates in response to changing circumstances that affect the relative costs and benefits of mating (Boulton & Shuker, 2016; Wilgers & Hebets, 2012).

There are two important fluctuating factors that are likely to regulate the direct benefits to female fertility of an additional mating. First is current female sperm limitation. Female insects have internal sperm storage organs where sperm are kept and used to fertilize eggs long after mating (Eberhard, 1996; Kotrba, 1995; Orr & Brennan, 2015; Pitnick, Markow, & Spicer, 1999). The current fertility status of a female will change over time; as females use up their sperm reserves or as sperm die, female fertility will probably decrease. Consequently, females that have mated recently or have full sperm storage organs will likely gain less benefit from an additional mating than sperm‐depleted females.

Second, the increase in female fertility from an additional mating may be influenced by the male's investment. Individual males have finite resources and their investment in ejaculates is predicted to be shaped by the trade‐off with the number of matings (Parker, 1982). There is good evidence that males increase their allocation to females that have higher reproductive value (Engqvist & Sauer, 2001; Kelly & Jennions, 2011; Perry, Sirot, & Wigby, 2013; Rogers, Grant, Chapman, Pomiankowski, & Fowler, 2006; Wedell et al., 2002). Likewise, in many situations, males increase their ejaculate size when females are subject to greater sperm competition (Kelly & Jennions, 2011; Wedell et al., 2002). It has been suggested that the quality of an ejaculate that a female receives may positively correlate with male condition (Iwasa & Pomiankowski, 1999; Sheldon, 1994), although firm evidence for this is lacking (Fitzsimmons & Bertram, 2013; Harley et al., 2013; Mautz, Møller, & Jennions, 2013; Pizzari, Jensen, & Cornwallis, 2004). Conversely, dominant or attractive males may invest fewer sperm per mating as they have more opportunities to mate and so need to divide their ejaculate into smaller packages per female (Jones, 2001; Tazzyman, Pizzari, Seymour, & Pomiankowski, 2009; Warner, Shapiro, Marcanato, & Petersen, 1995). In many cases, female fertility suffers when the male has recently mated (Levin, Mitra, & Davidowitz, 2016; Perez‐Staples, Aluja, Macías‐Ordóñez, & Sivinski, 2008; Torres‐Vila & Jennions, 2005; Wedell & Ritchie, 2004). The net effect is that female sperm limitation will vary with male mating strategy depending on the female's value to the male, the condition or attractiveness of the male, and his recent mating history. As a result, the direct fertility benefit that a female gains from an extra mating will not be a static quantity but will depend on the context in which mating takes place.

We examined how these two factors alter the benefits of female remating by means of experimentation in the wild using the Malaysian stalk‐eyed fly *Teleopsis dalmani* (Diptera, Diopsidae). Both sexes in this species are highly promiscuous (Wilkinson, Kahler, & Baker, 1998). Females typically have low fertility measured by egg hatch, both in the laboratory and in the wild (Baker et al., 2001; Cotton, Small, Hashim, & Pomiankowski, 2010). One of the main factors contributing to this infertility is that males have evolved to partition their ejaculates between many females. As a consequence, males transfer few sperm in a single copulation (~65, Wilkinson, Amitin, & Johns, 2005; ~142, Rogers et al., 2006) leading to females being sperm‐limited (Baker et al., 2001). Thus, females must remate in order to raise their fertility (Baker et al., 2001). As well as few sperm, the small size of male ejaculates is unlikely to provide any nonsperm benefits (Kotrba, 1996).

Given these patterns in stalk‐eyed flies, we expect to find that female *T. dalmanni* remate to gain direct fertility benefits. To distinguish between male and female effects as sources of variation in changes to female fertility, we report two experiments using wild‐caught *T. dalmanni* females. Prior mating histories of females and males cannot be controlled in field experiments. However, we initially kept females isolated from males in order that females became sperm‐depleted, to some extent. We then evaluated the effect of an additional mating on female fertility and expected that sperm‐depleted females should receive direct fertility benefits from an additional mating. To explore the impact of past male mating experience on the ability of males to confer fertility on females, in a second experiment we varied the prior mating rate and state of sperm depletion of wild‐caught males by keeping them for several days either with females or in male‐only groups. We then evaluated the fertility gain of females mated to these two types of male. These experiments allow us to examine, using wild‐caught individuals with backgrounds of natural variation, the extent of female and male effects on fertility.

## 2 MATERIALS AND METHODS

### 2.1 Experiment 1: Gains from an additional mating

Fly collections took place in February 2011 from eleven stream sites in the Ulu Gombak valley, Peninsular Malaysia (3°19′ N, 101°45′ E). Females and males were collected on day zero at dusk from lek sites on the edge of forest streams at several stream sites adjacent to tributaries of the Gombak River. Individuals were aspirated into plastic bags and within 1 hr of capture, males and females were transferred to individual 500‐ml containers lined with a moist cotton wool and tissue paper base. Flies were fed every 2 days with puréed banana.

Female fecundity was recorded from counts of eggs deposited on the tissue paper base, which were collected and renewed every 2 days. Eggs were allowed to develop for a further 5 days in petri dishes containing a moist cotton pad. Fertility was estimated by scoring hatching success under a light microscope at 10 ×  magnification. Fertilized eggs that have hatched appear as empty chorion cases, while unfertilized eggs are full and show no signs of development. If fertilized eggs failed to hatch, but showed signs of development (horizontal striations in the chorion and early mouthpart formation), they were recorded as fertile (Baker et al., 2001).

**Figure 1:**
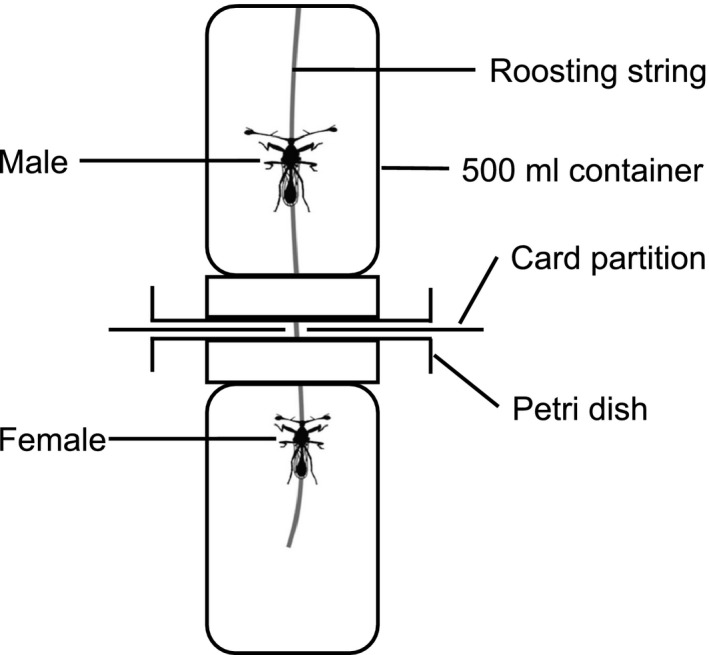
Mating chambers composed of two 500 ml cells, separated by a removable card partition. A single string runs the whole length of the chamber, providing a suitable roosting site. A male was placed in the upper cell and a female in the lower cell. The card partition was removed and the pair was allowed to mate once, before being separated

On day 13 after capture, each female was given a single additional mating with a male collected at the same time as the female. This time period was chosen to allow females to become sperm‐depleted prior to mating. Matings were carried out in mating chambers, each made up of two 500‐ml cells, separated by a removable card partition, and a single string running the length of the chamber provided a suitable roosting site (Cotton, Cotton, Small, & Pomiankowski, 2015; Figure 1). In the evening, a male was placed in the upper cell and the focal female in the lower cell. The following morning (after ~12 hr), the card partition was removed and the pair observed until a successful copulation took place, classed as lasting 30 s or more, to ensure that sperm transfer had occurred (Corley et al., 2006; Lorch, Wilkinson, & Reillo, 1993). Males were only used once. The remated females were then rehoused as before and their reproductive output was monitored from day 15 every 2 days for a further 8 days. The females were then killed and stored in ethanol. Female eyespan (distance between the outer tips of the eyes; Hingle, Fowler, & Pomiankowski, 2001) and thorax length (distance from base of the head to the joint between the metathoracic legs and the thorax; Rogers et al., 2008) were measured to an accuracy of 0.01 mm, using a monocular microscope and the image analysis software ImageJ, version 1.43e (Schneider, Rasband, & Eliceiri, 2012). In total, we recorded fertility for *N *= 45 females across the full sampling periods before and after the extra mating.

### 2.2 Experiment 2: Investigation of female and male effects

A second experiment was carried out using flies collected from five stream sites in the Ulu Gombak valley in July/August 2012. Individuals were collected as above. Females were housed individually in 500‐ml containers, and their reproductive output was recorded as in the first experiment. Males were placed in large 1,500‐ml containers either with a mix of males and nonfocal females allowing them to mate freely (sperm‐depleted), or only with other males (nonsperm‐depleted). Isolation from females allows males to replenish their sperm stores (Rogers, Chapman, Fowler, & Pomiankowski, 2005). Fly density was standardized across these two treatments, each pot containing a total of 10 flies, either a 1:1 ratio of males to females (sperm‐depleted) or 10 males (nonsperm‐depleted). On the evening of day 12, a focal female and male were placed in a mating container (Figure 1) and allowed to have an additional mating following the protocol above, except that males did not have an isolated overnight period. Females were placed either with a sperm‐depleted male (*N* = 19) or a nonsperm‐depleted male (*N* = 17). After the additional mating, females were rehoused and their subsequent reproductive output was recorded every 2 days from day 14 over the following 8 days, and morphometric measures taken as before.

### 2.3 Statistical analysis

Female sperm depletion was determined by the decline in female fertility over the 8 days before the single additional mating (comprising four egg counts) as well as over the 8 days after the additional mating (again, four egg counts). To test whether an additional mating resulted in increased fecundity or fertility, the total individual reproductive output over the 8 days before and after mating was compared, as well as total individual reproductive output on the days immediately before (days 11–12) and after the additional mating (days 14–15 in the first experiment; days 13–14 in the second experiment). Lastly, we examined whether the direction of change in individual fertility was positive, or negative/unchanged, and tested the degree to which individual proportion fertility changed depended on female premating fecundity or fertility.

All tests were carried out in R, version 3.31 (R Core Team, 2016), and are reported (including effect sizes) in the Appendix S1. Analyses were carried out of female reproductive output (fecundity and fertility), using generalized linear mixed‐effects models (GLMMs) using the *lme4* package (Bates, Mächler, Bolker, & Walker, 2015). Fecundity (number of eggs laid) and fertility (number of fertile eggs laid) were modeled in a GLMM with a Poisson distribution and log link function. In addition, egg counts were modeled as proportion data with a binomial distribution (fertile eggs, nonfertile eggs) and logit link function. We modeled the direction of change in individual fertility using a GLMM with a binomial distribution, where changes were coded as 1 s and 0 s (increase, decrease/unchanged). Change in proportion fertility (proportion after mating minus proportion before mating) was tested using a linear mixed‐effects model (LMM). Reported *p*‐values were computed by model comparison using ANOVA. Percentage fertility is described with the exclusion of females that laid fewer than 10 eggs.

Previous work showed a strong effect of stream site upon reproductive output (Harley, Fowler, & Cotton, 2010), so stream site was included as a random factor in reproductive output models—both in the first and second experiments. Variation between stream sites is reported for fecundity, fertility, and proportion fertility for the first experiment, where females were collected across 11 stream sites. They are not reported for the second experiment, as there was a more limited sample of only five stream sites, so any conclusions based on such a small sample would not be trustworthy. Where appropriate, female identity was included as a random factor to account for the nonindependence of multiple female measures. Variation between females is reported as a factor similar to stream sites.

The data were found to be overdispersed and to account for this, an observation‐level random effect (OLRE) was used in all models (except for those modeling change), as results can be unreliable when using both random effects and a quasi‐distribution (Harrison, 2014, 2015). The improvement in model fit from the addition of OLRE was checked through model comparison. OLRE may perform poorly in binomial models, so the parameter estimates of these models were checked against those from the comparable beta‐binomial model using the *glmmADMB* package (Fournier et al., 2012; Skaug, Fournier, Bolker, Magnusson, & Nielsen, 2016) to confirm robustness (Harrison, 2015).

Female eyespan and thorax length are known to be strong proxies for fecundity (Cotton, Fowler, & Pomiankowski, 2004; Rogers et al., 2006) and were highly correlated with female fecundity and fertility (Spearman's rank ρ > 0.3, *p* < .01). For both experiments, we repeated all analyses with female eyespan and thorax as covariates. This did not alter any of the results (see Appendix S1). For simplicity, the final models reported in the results did not include these covariates.

Reproductive output was examined over the 8 days before and 8 days after mating, excluding days 2 and 4 from all analyses. Previous studies have reported that reproductive output of recently caught *T. dalmanni* females typically falls in the short term (day 2) after mating, followed by a peak (day 4) before settling to a more steady level (Cotton et al., 2010; Harley et al., 2010). The same pattern was observed in this investigation (data not shown). Females that died or escaped during the observation period were excluded from the analyses (eight of 45 females in the first experiment; two of 36 females in the second experiment), as was a single female that failed to lay any eggs during the observation period in the first experiment.

## 3 RESULTS

### 3.1 Experiment 1: Gains from an additional mating

#### 3.1.1 Variation in fecundity

**Figure 2:**
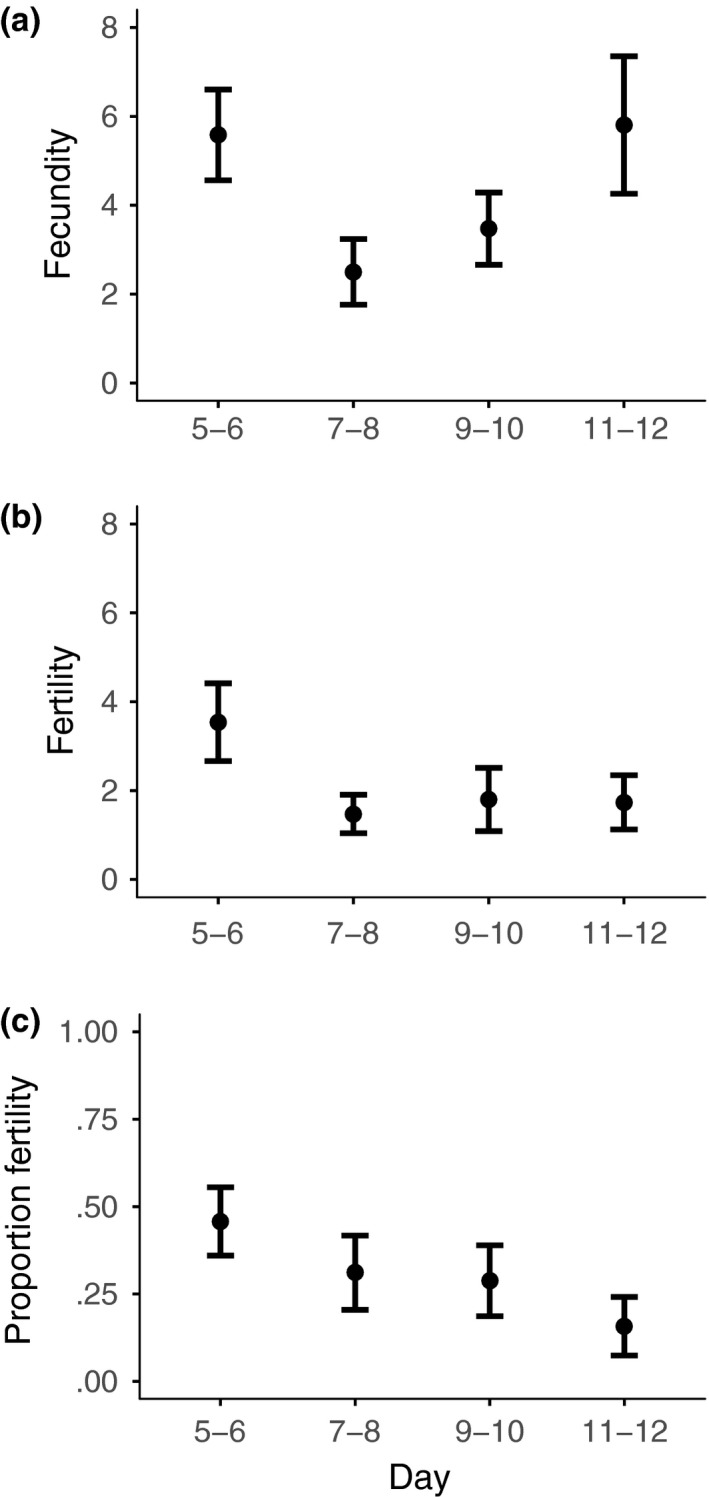
Premating female reproductive output through time (mean ± *SE*). Mean (a) fecundity, (b) fertility, and (c) proportion fertility per 2 days, over an 8‐day period. Flies were captured at dusk on day zero

Fecundity was highly variable between females both in the premating (days 5–12, mean ± *SD* per day = 2.17 ± 2.48; range = 0.13–11.13, *N* = 36; χ^2^ = 5.3291, *N *= 144, *p =* .0210) and postmating periods (days 14–21, mean ± *SD* per day = 2.42 ± 2.93, range = 0–11.88, *N* = 36; χ^2^ = 24.5018, *N *= 144, *p *< .0001). Female fecundity did not change over the premating period (χ^2^ = 1.1815, *N *= 144, *p =* .2770, Figure 2a), and there was no consistent directional change in fecundity over the whole 17‐day period of the experiment (χ^2^ = 1.2586, *N *= 288, *p =* .2619).

**Figure 3:**
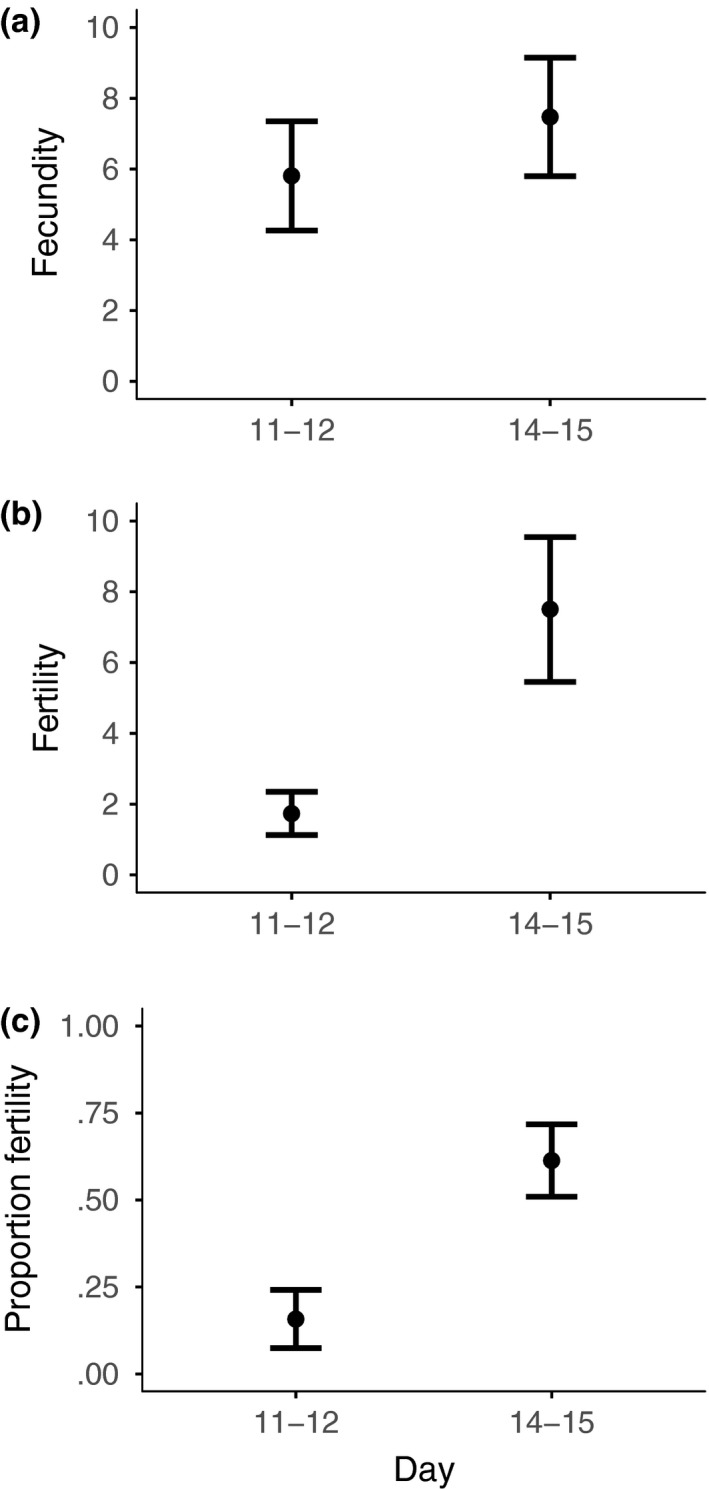
Reproductive output immediately before and immediately after mating (mean ± *SE*). Mean (a) fecundity, (b) fertility, and (c) proportion fertility on days 11–12 and days 14–15. Females were captured at dusk on day zero, and mating occurred at dawn on day 13

Female fecundity did not differ when individual reproductive output was compared across the premating and postmating periods (χ^2^ = 0.1001, *N *= 72, *p =* .7517), and was not different between the days immediately before (days 11–12) and immediately after (days 14–15) the extra mating (χ^2^ = 2.4907, *N *= 72, *p =* .1145, Figure 3a). Lastly, we examined differences in fecundity across streams. There was also no effect of stream site on fecundity in the premating (χ^2^ = 2.8652, *N *= 144, *p =* .0905) or postmating periods (χ^2^ = 0.0676, *N *= 144, *p =* .7948).

#### 3.1.2 Variation in fertility

The pattern for individual female fertility in the premating period (days 5–12), showed considerable variation among females, both in the absolute number of fertile eggs laid (mean ± *SD* per day = 0.66 ± 1.02; range = 0–4.75, *N* = 36; χ^2^ = 5.7493, *N *= 84, *p =* .0165) and proportion fertility (mean ± *SD* per day = 35.7057 ± 32.5241%, range = 0–86.3636%, *N* = 17; χ^2^ = 20.5766, *N *= 84, *p *< .0001), and this extended into the postmating period (days 14–21) for female absolute fertility (mean ± *SD* per day = 1.53 ± 2.59, range = 0–10.5, *N* = 36; χ^2^ = 9.7932, *N *= 85, *p =* .0018) but not proportion fertility (mean ± *SD* = 58.5037% ± 33.0920%, range = 0–100%, *N* = 21; χ^2^ = 3.4542, *N *= 85, *p =* .0631). In contrast to fecundity, across the premating period there was a decline in absolute (χ^2^ = 8.4502, *N *= 84, *p *= .0037, Figure 2b) and proportion fertility (χ^2^ = 17.5402, *N *= 84, *p <* .0001, Figure 2c). Note that it was important to examine proportion fertility as there was a positive relationship between total female fertility and fecundity both in the premating (χ^2^ = 5.9894, *N *= 36, *p =* .0144) and postmating periods (χ^2^ = 22.6367, *N *= 32, *p <* .0001).

**Figure 4:**
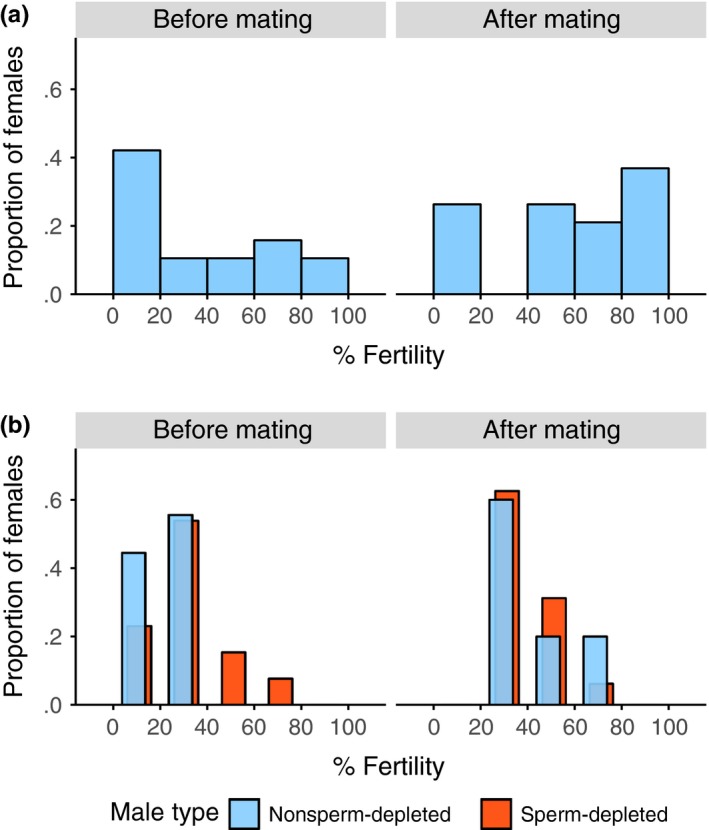
The distribution of percentage fertility (total eggs hatched / total eggs laid) for females in the 8 days before, and 8 days after the extra mating in (a) experiment 1 and (b) experiment 2. Females used in experiment 2 were either mated to a sperm‐depleted (orange) or a nonsperm‐depleted male (light blue). Plots exclude females who laid fewer than 10 eggs over each period

Comparing total fertility over the whole premating and postmating periods, absolute fertility did not change after the additional mating (χ^2^ = 3.5892, *N = *68, *p =* .0582); however, proportion fertility increased (χ^2^ = 5.1530, *N = *68, *p =* .0232). The percentage of females with low fertility (<20% total egg hatch) dropped from 38% to 19%, whereas the proportion with high fertility (>70% total egg hatch) rose from 24% to 48% (Figure 4a). Comparing across a closer period of time, there was a distinct increase in the days around the extra mating (days 11–12 to days 14–15), both absolute (χ^2^ = 10.0766, *N =* 41, *p = *.0015, Figure 3b) and proportion fertility increased (χ^2^ = 15.5344, *N =* 41, *p < *.0001, Figure 3c).

The direction of change in total individual fertility after the additional mating (increase or decrease/unchanged) did not depend on female fecundity (χ^2^ = 2.2001, *N =* 32, *p *= .1380). However, when female fertility was accounted for, females with higher fecundity were more likely to have a positive change in fertility after the additional mating (χ^2^ = 18.3375, *N =* 32, *p *< .0001). In addition, females with low fertility were more likely to benefit from the additional mating (χ^2^ = 5.8261, *N =* 32, *p *= .01579). This greater effect of premating fertility persisted after accounting for differences in individual female fecundity (χ^2^ = 21.9635, *N =* 32, *p *< .001).

**Figure 5:**
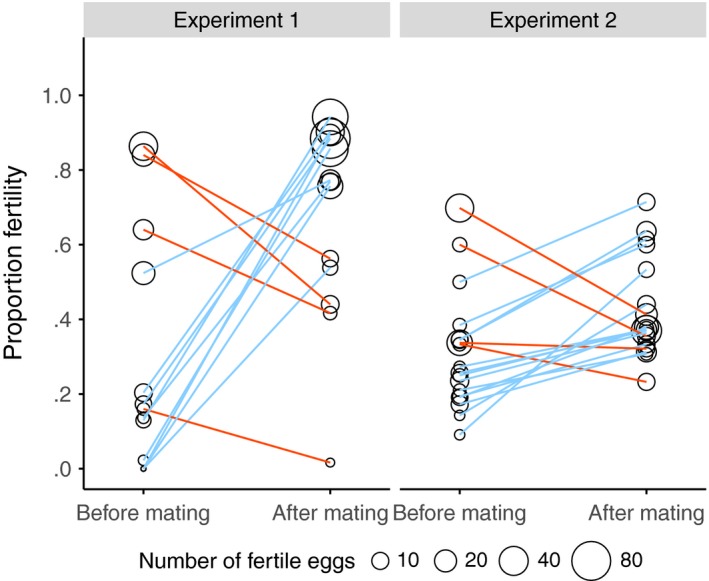
Total proportion fertility in the 8 days before, and 8 days after the extra mating in experiment 1 and experiment 2. Lines are individual females, colored by slope: increased fertility (light blue), decreased fertility (orange). Circle size indicates the total absolute number of fertile eggs laid by each female. Plots exclude females that laid fewer than 10 eggs either before or after the extra mating

A similar examination was made using the change in proportion fertility between the pre‐ and postmating periods (Figure 5). Females with high premating fecundity had a larger positive change in their proportion fertility postmating (χ^2^ = 7.5575, *N =* 32, *p *= .0060), and this result remained when female fertility was accounted for (χ^2^ = 12.842, *N =* 32, *p *< .0001). Female premating fertility had no effect on the change in proportion fertility (χ^2^ = 2.0648, *N *= 32, *p *= .1507). However, once fecundity was accounted for, female premating fertility did have an effect (χ^2^ = 7.349, *N =* 32, *p *= .0067), as females that fertilized few of their eggs had a larger positive change in proportion fertility than females that were already fertilizing relatively more.

Finally, we examined differences in fertility across streams. In the premating period, there was variation between stream sites in absolute (χ^2^ = 5.8958, *N *= 84, *p =* .0152) and proportion fertility (χ^2^ = 4.3233, *N *= 84, *p =* .0376). After the additional mating, absolute fertility no longer differed between stream sites (χ^2^ = 1.4439, *N *= 85, *p *= .2295), but variation in proportion fertility persisted despite the extra mating (χ^2^ = 5.5951, *N *= 85, *p *= .0180).

### 3.2 Experiment 2: Investigation of female and male effects

To investigate potential male effects on fertility gain among females, a second experiment was carried out. Females were mated once either with a sperm‐depleted male that had been held for the previous 2 weeks with multiple females or with a nonsperm‐depleted male that had been held in a male‐only container.

#### 3.2.1 Variation in fecundity

**Figure 6:**
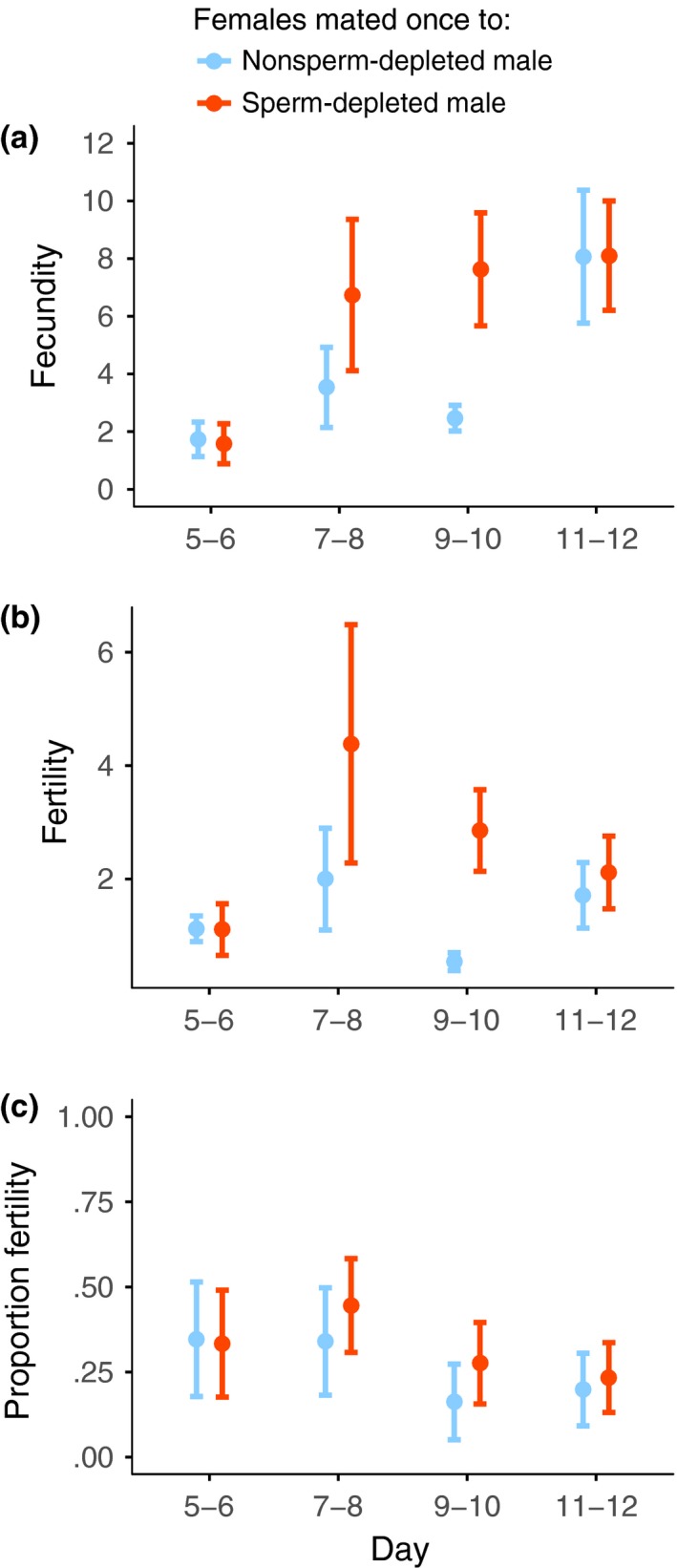
Premating female reproductive output of mean (a) fecundity, (b) fertility, and (c) proportion fertility per 2 days through time (mean ± *SE*). Females from the sperm‐depleted (orange) or nonsperm‐depleted male (light blue) treatment are shown separately. Flies were captured at dusk on day zero

**Figure 7:**
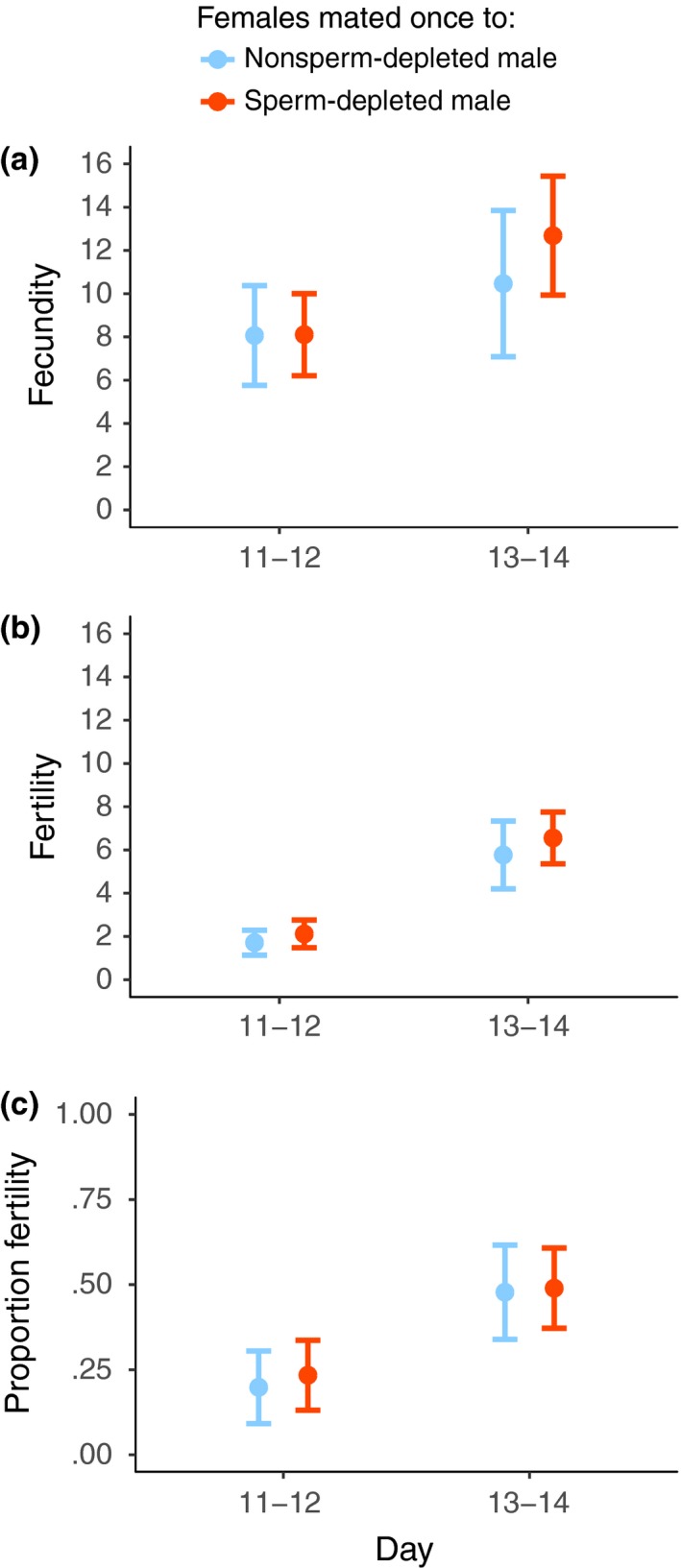
Reproductive output immediately before and immediately after mating (mean ± *SE*) where females received an extra mating from either a sperm‐depleted (orange) or nonsperm‐depleted (light blue) male. Mean (a) fecundity, (b) fertility, and (c) proportion fertility on days 11–12 and days 13–14. Females were captured at dusk on day zero and mating occurred on the evening of day 12

The pattern for female fecundity was broadly similar to that of the previous experiment (Figure 6a and 7a, see Appendix S1). There was no effect of male type on total fecundity before versus after the additional mating (male type × before/after interaction, χ^2^ = 0.4838, *N *= 68, *p *= .4867), or for the contrast of the days immediately before and after the additional mating, days 11–12 and 13–14 (χ^2^ = 0.5267, *N *= 68, *p* = .4680).

#### 3.2.2 Variation in fertility

Fertility also showed a broadly similar pattern to the previous experiment (Figure 4b, 6b and 7b, see Appendix S1). At the end of the premating period, individual absolute fertility was comparable to that of the low absolute fertility in the previous experiment (1.7368 ± 2.6634 and 1.9355 ± 2.4074, expt. 1 and expt. 2, mean ± *SD*, days 11–12). Proportion fertility was also similar to the previous experiment prior to mating (19% and 21%, expt. 1 and expt. 2, days 11–12). Comparing total fertility in the premating and postmating periods, absolute (χ^2^ = 12.5805, *N *= 66, *p *< .0001) and proportion fertility (χ^2^ = 12.4228, *N *= 66, *p *< .0001) increased after the additional mating. Likewise, between the days immediately prior (day 11–12) and immediately after (days 13–14) the additional mating, there was an increase in absolute (χ^2^ = 23.8148, *N *= 62, *p *< .0001, Figure 7b) and proportion fertility (χ^2^ = 27.0669, *N *= 62, *p *< .0001, Figure 7c).

The direction of change in individual fertility was more likely to be positive for more fecund females (χ^2^ = 4.7193, *N =* 32, *p *= .0298), but not after female fertility was accounted for (χ^2^ = 0.1939, *N =* 32, *p *= .6597). Females with low premating fertility were more likely to have a positive change (χ^2^ = 8.2079, *N =* 32, *p *= .0042). However again, after accounting for fecundity, premating fertility did not predict the direction of change (χ^2^ = 3.6824, *N =* 32, *p *= .0550).

Change in proportion fertility between the premating and postmating periods did not depend on premating fecundity (χ^2^ = 0.0476, *N =* 32, *p *= .8274, Figure 5), but when female fertility was controlled for, more fecund females had a more positive change in proportion fertility (χ^2^ = 4.4386, *N =* 32, *p *= .0351). Change in proportion fertility likewise did not depend on premating fertility (χ^2^ = 3.1064, *N *= 32, *p *= .0780). In addition, when the analysis was repeated and fecundity was accounted for, females with low fertility prior to mating also had a more positive change in proportion fertility (χ^2^ = 7.4975, *N =* 32, *p *= .0062).

Comparing the 8 days before and after the additional mating, male type was unrelated to the increase in absolute (male type × before/after interaction, χ^2^ = 0.6327, *N *=* *66, *p *=* *.4264) and proportion fertility (χ^2^ = 2.6744, *N *=* *66, *p *=* *.1020). Likewise comparing the days immediately before (day 12) and after the additional mating (day 14), male type had no effect on the increase in absolute (χ^2^ = 0.0027, *N *=* *62, *p *=* *.9589) or proportion fertility (χ^2^ = 0.2317, *N *=* *62, *p *=* *.6303). There was no effect of male type on either the direction of change in fertility (χ^2^ = 0.2076, *N = *32, *p *=* *.6487) or the change in proportion fertility (χ^2^ = 0.4654, *N = *32, *p *=* *.4951).

## 4 DISCUSSION

There are abundant studies investigating the direct fertility benefits from multiple mating (Arnqvist & Nilsson, 2000; Haig & Bergstrom, 1995; Hosken & Stockley, 2003; Slatyer et al., 2012; Yasui, 1998; Zeh & Zeh, 1996). However, there is currently minimal focus on how these benefits vary between individuals and across time, or in particular contexts like associations with the degree of polyandry and female age or experience (House et al., 2009; Toft & Albo, 2015; Wright et al., 2013). In addition, experiments evaluating direct benefits of multiple mating have rarely been carried out among individuals sampled from wild populations, in ways that examine the encounters likely to occur between females and males in nature.

In this study, we aimed to redress these deficits by assessing fecundity and fertility in wild‐caught stalk‐eyed flies, and how these benefits vary with the time since the last mating (and, as a corollary, whether there is a cost of a failure to remate that increases with time). Females from laboratory populations of *T. dalmanni* have been shown to benefit from multiple mating (Baker et al., 2001). But the experience of flies under laboratory conditions is inevitably very different from those in wild populations, for example, in terms of population density, food availability, and exposure to parasites/predators. Moreover, laboratory studies of stalk‐eyed flies and other species have utilized virgin males and females in remating assays, in order to standardize prior mating experience (Baker et al., 2001; Bayoumy, Michaud, & Bain, 2015; Burdfield‐Steel, Auty, & Shuker, 2015; Chelini & Hebets, 2016; Droge‐Young, Belote, Eeswara, & Pitnick, 2016; Tregenza & Wedell, 2002). But virgins are rare in nature in species in which males and females readily remate, and this is particularly true of stalk‐eyed flies in which adult fertility persists for many weeks (Rogers et al., 2006). All of these factors point to the necessity for controlled experiments using wild‐caught individuals with backgrounds of natural variation.

Female sperm limitation is likely to be an important fluctuating factor that regulates the direct fertility benefits to females from multiple mating. In some insect mating systems females only mate once (Arnqvist & Andrés, 2006; Arnqvist & Nilsson, 2000; South & Arnqvist, 2008) or mate multiple times but over a single short period (Boomsma, Baer, & Heinze, 2005). These restricted mating patterns provide sufficient sperm to ensure female fertility throughout her reproductive life. However, in many other insect species, sperm acquired in a single mating or mating period is insufficient to fertilize all her eggs (Ridley, 1988; Wedell et al., 2002). Consequently, females necessarily need to remate throughout their adult life, as sperm supplies diminish through use and with time (Chevrier & Bressac, 2002; Drnevich et al., 2001; Fjerdingstad & Boomsma, 1998; Fox, 1993; Wang & Davis, 2006). We demonstrate that this form of reproductive life history typifies *T. dalmanni* stalk‐eyed fly females collected from the wild. Females from the two collections, in 2011 and 2012, had mean female fertility of 46% or 32%, respectively, shortly after they were initially captured (days 5–6), and this declined to ~20% in both cases over the following week (days 11–12; Figures 2 and 6). An additional mating after 12 days markedly changed fertility, causing a substantially larger proportion of their eggs to be fertilized, 61% and 48%, immediately after the additional mating (Figures 3 and 7). In contrast, female fecundity was unchanged by an additional mating and remained consistent across the whole of the study period, although with a fair degree of stochastic variation (Figures 2 and 6). Accordingly, negative and positive changes in fertility can be ascribed to females being able to fertilize a smaller or larger proportion of their eggs, rather than due to fluctuations in the number of eggs laid.

We show an overall increase in fertility; however, we additionally make the novel finding that the increase in fertility was not uniform between individual females. Females with low premating fertility were more likely to benefit from an additional mating, as were females with high fecundity. After taking account of variation in premating fecundity, it is apparent that females were able to fertilize a larger proportion of their eggs if they initially had low fertility. Similarly, after taking account of variation in premating fertility, females gained more in fertility from an additional mating if they were highly fecund. These outcomes reveal a strong context dependence in the benefit of additional matings. Low prior fertility is indicative that females were subject to sperm depletion, and high fecundity is indicative of the need for greater numbers of stored sperm, both seemingly addressed by the additional mating. To test these predictions, direct measurements of sperm numbers within females will be necessary. This is possible in female stalk‐eyed flies, which retain sperm in spermathecae that act as long‐term storage organs, and the ventral receptacle, a small structure to which sperm move and are stored individually within pouches (capacity ~16–40 sperm) prior to release for fertilization of an egg (Kotrba, 1993; Rose, Brand, & Wilkinson, 2014).

The results here contrast with those of a previous study carried out on the same population (Harley et al., 2010). In that study, females were collected from the wild at lek mating sites and half were immediately allowed a single additional mating. Both groups showed a decline in fertility through time, as in the current study. However, there was no difference in fertility between females that received an extra mating on capture and those that did not. What explains the divergence from the current study? The striking difference is that females were unusually fertile, ~80% over the first 10 days in captivity, both among females with and females without the extra mating (Harley et al., 2010). This degree of fertility is comparable to the levels achieved in laboratory populations when females are given the opportunity to mate repeatedly (Baker et al., 2001). This failure of an additional mating to enhance female fertility echoes our finding that fertility gains from an extra mating are weaker when females already have high fertility. In the current study, average fertility was much lower, around ~30% fertility in both years of this study. Hence, there was plenty of opportunity for an extra mating to benefit female fertility. We suspect this low level is the norm as an earlier census also from the same area in Malaysia reported 36% fertility (Cotton et al., 2010).

We can make several inferences from these studies of wild‐caught females. First, they confirm there is a cost of a failure to remate as the proportion of fertile eggs laid declines with time when females are unable to remate. Second, an additional mating has a greater beneficial effect when females already have low fertility. The most obvious proximate reason for this is that many wild females are sperm‐limited, either because they had not mated recently, not mated at a sufficiently high rate or because sperm allocation by males was considerably limited. These explanations could be directly assessed in the future by counting sperm in female sperm storage organs in wild‐caught females and after matings with wild‐caught males. This could be complemented by observing mating rates in the wild, and relating these measures to natural fertility levels. A third inference from the current experiments is that the fertility benefits to females vary between individuals, stream sites, across matings and fluctuate through time. In some contexts, individual females may be limited by the availability of mating opportunities, whereas in others, they may become increasingly limited by their own fecundity.

The source of variation in fertility between individuals in the wild is currently undefined. It is likely that variable factors such as population density and sex ratio are important, particularly as they will affect female and male mating rates. Similarly, environmental conditions such as food availability can influence mating rates (Kotiaho, Simmons, & Tomkins, 2001; Rogers et al., 2005, 2008), male fertility (Bunning et al., 2015; O'Dea, Jennions, & Head, 2014; Perry & Rowe, 2010; Perry et al., 2013) and female fecundity (Awmack & Leather, 2002; Cotton et al., 2015; Levin et al., 2016; Stewart, Morrow, & Rice, 2005). While in certain contexts an additional mating may be clearly beneficial for female fertility, we show that this is not always the case and there is a need to test females under a range of contexts that reflect those experienced under natural conditions. Only then can the full force of remating on female fertility be understood.

Other significant factors to consider are variation in male mating strategy and male quality as they may have a significant influence on the benefit that females obtain from remating. Males can adjust their ejaculate investment in response to female reproductive value (Engqvist & Sauer, 2001; Kelly & Jennions, 2011; Perry et al., 2013; Rogers et al., 2006; Wedell et al., 2002) and sperm competition (Kelly & Jennions, 2011; Wedell et al., 2002), and investment may positively correlate with male condition (Iwasa & Pomiankowski, 1999; Sheldon, 1994; but see Fitzsimmons & Bertram, 2013; Harley et al., 2013; Mautz et al., 2013; Pizzari et al., 2004) or negatively with male dominance or attractiveness (Jones, 2001; Tazzyman et al., 2009; Warner et al., 1995). We explicitly evaluated the importance of variation in recent male mating experience, contrasting males that had multiple opportunities to mate, with those that had been deprived of females. Rather surprisingly, there was no difference in fertility gains from extra matings with either type of male (Figure 7b,c). This reveals that male allocation of ejaculate is tailored to repeated mating, and the replenishment of resources occurs on a short time scale. Males partition their ejaculate in order to copulate with many females each day (Small, Cotton, Fowler, & Pomiankowski, 2009); spermatophore size is very small in *T. dalmanni* (Kotrba, 1996), and males transfer few sperm in a single ejaculate (~100, Rogers et al., 2006; Wilkinson et al., 2005). Partitioning of ejaculate is presumably a mechanism for males to maintain fertility over successive matings (Linklater, Wertheim, Wigby, & Chapman, 2007; Wedell et al., 2002). In addition, male reproductive activity is scheduled in a highly concentrated burst each day, as lek‐holding males mate with females that have settled with them overnight before they disperse at dawn (Chapman, Pomiankowski, & Fowler, 2005; Cotton et al., 2010). To cope with this pattern of sexual activity, males replenish their accessory glands and hence their ability to produce ejaculate within 24 hr (Rogers et al., 2005). In this system, prior mating activity has no or a minimal effect on a male's ability to mate effectively. However, we only assessed female fertility gains after the first mating by a male. It might still be the case that prior mating experience could affect the ability of males to deliver ejaculate in subsequent matings or even to be able to mate repeatedly. In the wild, it is notable that females often leave lek sites before mating if the male is pre‐occupied in matings with other females (A. Pomiankowski, personal observation). This suggests that fertility gains may fall with subsequent matings, but this remains to be investigated. Again, this points to the complexity of context underpinning the benefits associated with remating.

Another cause of variation in male fertility and ejaculate allocation, other than recent mating history, is meiotic drive (Wilkinson, Johns, Kelleher, Muscedere, & Lorsong, 2006). An X‐linked meiotic drive system is present in these populations of *T. dalmanni* (Cotton, Földvári, Cotton, & Pomiankowski, 2014) and causes the degeneration of Y‐bearing sperm and the production of female‐biased broods (Presgraves, Severance, & Wilkinson, 1997). We expect drive male fertility to be reduced due to this dysfunction resulting in the transfer of fewer sperm. Consequently, mating with a drive male may not provide a female with the same fertility benefit as mating with a standard male. There is evidence that females mated to drive males have lower fertility, particularly when males are mating at high frequencies (Wilkinson, Swallow, Christianson, & Madden, 2003; Wilkinson et al., 2006) and that drive males are poor sperm competitors (Wilkinson et al., 2006). In this study, we found that several females failed to raise their fertility after mating (Figure 5), and in fact had lower fertility than prior to mating. Mating with a drive male could potentially produce this pattern. Future research should evaluate explicitly how an extra mating with a drive male impacts on female fertility among wild‐caught flies, when males and females are in their natural condition. It would also be of interest to investigate the hypothesis that multiple mating is an evolved mechanism by which females dilute the negative effects of mating with a drive male (Haig & Bergstrom, 1995; Zeh & Zeh, 1996), both to ensure fertility and because any male progeny produced in a female‐biased population will have increased fitness (Fisher, 1930; Holman, Price, Wedell, & Kokko, 2015).

We used wild‐caught flies to capture the natural variation between individuals, an approach that has been much neglected. It is important to dig deeper into the life history of *T. dalmanni* to further understand the environmental and population‐level variables that affect the benefits to additional matings. For example, we know that there is much variation in female fecundity and fertility between stream sites. What we have yet to elucidate is how streams differ—do they vary in food availability and quality, rainfall, humidity, temperature, population density, or sex ratio? Are these factors stable or fluctuating? Which have the most influence on female fecundity and fertility? We show that females with low fertility and high fecundity benefit the most from mating; improved knowledge of the conditions experienced by individuals throughout their lifetime will further our understanding of when and why it is beneficial for females to remate.

In conclusion, this study has demonstrated that female sperm storage and depletion since the previous mating are key selection forces driving the benefits and evolution of mating rates in the wild. Females are generally sperm‐limited due to the minimal male sperm investment in individual copulations (Rogers et al., 2006; Wilkinson et al., 2005), so females gain direct fertility benefits from multiple mating both in the laboratory (Baker et al., 2001) and in wild populations. However, these gains are not uniform between females and are contingent on female fecundity and fertility. In a broader context, stalk‐eyed fly reproductive activity is governed by a co‐evolutionary spiral of exaggerated mating rates. Females have evolved high levels of multiple mating because their fertility is subject to sperm limitation. The resulting higher levels of multiple mating by males, especially those that are attractive to females, have led to the evolutionary corollary of finer partitioning of ejaculate, which has only exacerbated sperm limitation and the benefits of multiple mating. The various studies of stalk‐eyed fly fertility in the wild (Cotton et al., 2010; Harley et al., 2010; this study) demonstrate both high variation (across space and time, and between individuals) and now also context dependence in benefits to remating. They highlight the importance of complementing laboratory studies with those using wild populations, where natural mating rates may be very different. Further studies will disentangle whether other factors such as variation in age, condition, attractiveness, a range of environmental variables, and the presence of meiotic drive are important as well, and allow a better understanding of the range of forces that influence female and male mating behavior.

## CONFLICT OF INTEREST

None declared.

## AUTHORS’ CONTRIBUTIONS

EH, AP, and KF conceived the original project and methodology; EH, AC, and JMH collected the data; LM analyzed the data; LM, AP, and KF led the writing of the manuscript. All authors contributed critically to the drafts and gave final approval for publication.

## DATA ACCESSIBILITY

Raw data have been archived in the Dryad Digital Repository: https://doi.org/10.5061/dryad.pk5vd.

## References

[ece33486-bib-0001] Arnqvist, G. , & Andrés, J. A. (2006). The effects of experimentally induced polyandry on female reproduction in a monandrous mating system. Ethology, 112(8), 748–756. https://doi.org/10.1111/j.1439-0310.2006.01211.x

[ece33486-bib-0002] Arnqvist, G. , & Nilsson, T. (2000). The evolution of polyandry: Multiple mating and female fitness in insects. Animal Behaviour, 60(2), 145–164. https://doi.org/10.1006/anbe.2000.1446 10973716 10.1006/anbe.2000.1446

[ece33486-bib-0003] Avise, J. C. , Jones, A. G. , Walker, D. , & DeWoody, J. A. (2002). Genetic mating systems and reproductive natural histories of fishes: Lessons for ecology and evolution. Annual Review of Genetics, 36(1), 19–45. https://doi.org/10.1146/annurev.genet.36.030602.090831 10.1146/annurev.genet.36.030602.090831 12429685

[ece33486-bib-0004] Awmack, C. S. , & Leather, S. R. (2002). Host plant quality and fecundity in herbivorous insects. Annual Review of Entomology, 47(1), 817–844. https://doi.org/10.1146/annurev.ento.47.091201.145300 10.1146/annurev.ento.47.091201.145300 11729092

[ece33486-bib-0005] Baker, R. H. , Ashwell, R. , Richards, T. , Fowler, K. , Chapman, T. , & Pomiankowski, A. (2001). Effects of multiple mating and male eye span on female reproductive output in the stalk‐eyed fly, *Cyrtodiopsis dalmanni* . Behavioral Ecology, 12(6), 732–739. https://doi.org/10.1093/beheco/12.6.732

[ece33486-bib-0006] Bateman, A. J. (1948). Intra‐sexual selection in *Drosophila* . Heredity, 2(3), 349–368. https://doi.org/10.1038/hdy.1948.21 18103134 10.1038/hdy.1948.21

[ece33486-bib-0007] Bates, D. , Mächler, M. , Bolker, B. , & Walker, S. (2015). Fitting linear mixed‐effects models using *lme4* . Journal of Statistical Software, 67(1), 1–48. https://doi.org/10.18637/jss.v067.i01

[ece33486-bib-0008] Bayoumy, M. H. , Michaud, J. P. , & Bain, C. (2015). Polyandry restores female fertility and paternal effects diminished by inbreeding in *Hippodamia convergens* . Ecological Entomology, 40(5), 596–602. https://doi.org/10.1111/een.12230

[ece33486-bib-0009] Boomsma, J. J. , Baer, B. , & Heinze, J. (2005). The evolution of male traits in social insects. Annual Review of Entomology, 50(1), 395–420. https://doi.org/10.1146/annurev.ento.50.071803.130416 10.1146/annurev.ento.50.071803.130416 15822204

[ece33486-bib-0010] Boulton, R. A. , & Shuker, D. M. (2016). Polyandry is context dependent but not convenient in a mostly monandrous wasp. Animal Behaviour, 112, 119–125. https://doi.org/10.1016/j.anbehav.2015.12.001

[ece33486-bib-0011] Bunning, H. , Rapkin, J. , Belcher, L. , Archer, C. R. , Jensen, K. , & Hunt, J. (2015). Protein and carbohydrate intake influence sperm number and fertility in male cockroaches, but not sperm viability. Proceedings of the Royal Society B: Biological Sciences, 282(1802), 20142144 https://doi.org/10.1098/rspb.2014.2144 25608881 10.1098/rspb.2014.2144 PMC4344140

[ece33486-bib-0012] Burdfield‐Steel, E. R. , Auty, S. , & Shuker, D. M. (2015). Do the benefits of polyandry scale with outbreeding? Behavioral Ecology, 26(5), 1423–1431. https://doi.org/10.1093/beheco/arv103 26379413 10.1093/beheco/arv103 PMC4568444

[ece33486-bib-0013] Chapman, T. , Pomiankowski, A. , & Fowler, K. (2005). Stalk‐eyed flies. Current Biology, 15(14), R533–R535. https://doi.org/10.1016/j.cub.2005.07.015 16051154 10.1016/j.cub.2005.07.015

[ece33486-bib-0014] Chelini, M. C. , & Hebets, E. A. (2016). Polyandry in the absence of fitness benefits in a species with female‐biased sexual size dimorphism. Animal Behaviour, 119, 213–222. https://doi.org/10.1016/j.anbehav.2016.07.008

[ece33486-bib-0015] Chevrier, C. , & Bressac, C. (2002). Sperm storage and use after multiple mating in *Dinarmus basalis* (Hymenoptera: Pteromalidae). Journal of Insect Behavior, 15(3), 385–398. https://doi.org/10.1023/A:1016269210140

[ece33486-bib-0016] Clutton‐Brock, T. H. (1989). Review lecture: Mammalian mating systems. Proceedings of the Royal Society B: Biological Sciences, 236(1285), 339–372. https://doi.org/10.1098/rspb.1989.0027 10.1098/rspb.1989.0027 2567517

[ece33486-bib-0017] Cordero, C. , & Eberhard, W. G. (2003). Female choice of sexually antagonistic male adaptations: A critical review of some current research. Journal of Evolutionary Biology, 16(1), 1–6. https://doi.org/10.1046/j.1420-9101.2003.00506.x 14635875 10.1046/j.1420-9101.2003.00506.x

[ece33486-bib-0018] Corley, L. S. , Cotton, S. , McConnell, E. , Chapman, T. , Fowler, K. , & Pomiankowski, A. (2006). Highly variable sperm precedence in the stalk‐eyed fly, *Teleopsis dalmanni* . BMC Evolutionary Biology, 6, 53 https://doi.org/10.1186/1471-2148-6-53 16800877 10.1186/1471-2148-6-53 PMC1543661

[ece33486-bib-0019] Cotton, A. J. , Cotton, S. , Small, J. , & Pomiankowski, A. (2015). Male mate preference for female eyespan and fecundity in the stalk‐eyed fly, *Teleopsis dalmanni* . Behavioral Ecology, 26(2), 376–385. https://doi.org/10.1093/beheco/aru192

[ece33486-bib-0020] Cotton, A. J. , Földvári, M. , Cotton, S. , & Pomiankowski, A. (2014). Male eyespan size is associated with meiotic drive in wild stalk‐eyed flies (*Teleopsis dalmanni*). Heredity, 112(4), 363–369. https://doi.org/10.1038/hdy.2013.131 24398884 10.1038/hdy.2013.131 PMC3966131

[ece33486-bib-0021] Cotton, S. , Fowler, K. , & Pomiankowski, A. (2004). Condition dependence of sexual ornament size and variation in the stalk‐eyed fly *Cyrtodiopsis dalmanni* (Diptera: Diopsidae). Evolution, 58(5), 1038–1046. https://doi.org/10.1111/j.0014-3820.2004.tb00437.x 15212384 10.1111/j.0014-3820.2004.tb00437.x

[ece33486-bib-0022] Cotton, S. , Small, J. , Hashim, R. , & Pomiankowski, A. (2010). Eyespan reflects reproductive quality in wild stalk‐eyed flies. Evolutionary Ecology, 24(1), 83–95. https://doi.org/10.1007/s10682-009-9292-6

[ece33486-bib-0023] Crean, A. J. , & Marshall, D. J. (2009). Coping with environmental uncertainty: Dynamic bet hedging as a maternal effect. Philosophical Transactions of the Royal Society B: Biological Sciences, 364(1520), 1087–1096. https://doi.org/10.1098/rstb.2008.0237 10.1098/rstb.2008.0237 PMC2666679 19324613

[ece33486-bib-0024] Drnevich, J. M. , Papke, R. S. , Rauser, C. L. , & Rutowski, R. L. (2001). Material benefits from multiple mating in female mealworm beetles (*Tenebrio molitor* L.). Journal of Insect Behavior, 14(2), 215–230. https://doi.org/10.1023/A:1007889712054

[ece33486-bib-0025] Droge‐Young, E. M. , Belote, J. M. , Eeswara, A. , & Pitnick, S. (2016). Extreme ecology and mating system: Discriminating among direct benefits models in red flour beetles. Behavioral Ecology, 27(2), 575–583. https://doi.org/10.1093/beheco/arv191

[ece33486-bib-0026] Eberhard, W. G. (1996). Female control: Sexual selection by cryptic female choice. Princeton: Princeton University Press.

[ece33486-bib-0027] Engqvist, L. , & Sauer, K. P. (2001). Strategic male mating effort and cryptic male choice in a scorpionfly. Proceedings of the Royal Society B: Biological Sciences, 268(1468), 729–735. https://doi.org/10.1098/rspb.2000.1423 11321062 10.1098/rspb.2000.1423 PMC1088663

[ece33486-bib-0028] Fisher, R. A. (1930). The genetical theory of natural selection: A complete variorum edition. Oxford: Oxford University Press.

[ece33486-bib-0029] Fitzsimmons, L. P. , & Bertram, S. M. (2013). No relationship between long‐distance acoustic mate attraction signals and male fertility or female preference in spring field crickets. Behavioral Ecology and Sociobiology, 67(6), 885–893. https://doi.org/10.1007/s00265-013-1511-z

[ece33486-bib-0030] Fjerdingstad, E. J. , & Boomsma, J. J. (1998). Multiple mating increases the sperm stores of *Atta colombica* leafcutter ant queens. Behavioral Ecology and Sociobiology, 42(4), 257–261. https://doi.org/10.1007/s002650050437

[ece33486-bib-0031] Fournier, D. A. , Skaug, H. J. , Ancheta, J. , Ianelli, J. , Magnusson, A. , Maunder, M. N. , … Sibert, J. (2012). AD Model Builder: Using automatic differentiation for statistical inference of highly parameterized complex nonlinear models. Optimization Methods and Software, 27(2), 233–249. https://doi.org/10.1080/10556788.2011.597854

[ece33486-bib-0032] Fox, C. W. (1993). Multiple mating, lifetime fecundity and female mortality of the bruchid beetle, *Callosobruchus maculatus* (Coleoptera: Bruchidae). Functional Ecology, 7(2), 203–208. https://doi.org/10.2307/2389888

[ece33486-bib-0033] Ginsberg, J. R. , & Huck, U. W. (1989). Sperm competition in mammals. Trends in Ecology & Evolution, 4(3), 74–79. https://doi.org/10.1016/0169-5347(89)90152-3 21227319 10.1016/0169-5347(89)90152-3

[ece33486-bib-0034] Griffith, S. C. , Owens, I. P. F. , & Thuman, K. A. (2002). Extra pair paternity in birds: A review of interspecific variation and adaptive function. Molecular Ecology, 11(11), 2195–2212. https://doi.org/10.1046/j.1365-294X.2002.01613.x 12406233 10.1046/j.1365-294x.2002.01613.x

[ece33486-bib-0035] Haig, D. , & Bergstrom, C. T. (1995). Multiple mating, sperm competition and meiotic drive. Journal of Evolutionary Biology, 8(3), 265–282. https://doi.org/10.1046/j.1420-9101.1995.8030265.x

[ece33486-bib-0036] Harley, E. , Birge, L. M. , Small, J. , Tazzyman, S. J. , Pomiankowski, A. , & Fowler, K. (2013). Ejaculate investment and attractiveness in the stalk‐eyed fly, *Diasemopsis meigenii* . Ecology and Evolution, 3(6), 1529–1538. https://doi.org/10.1002/ece3.544 23789065 10.1002/ece3.544 PMC3686189

[ece33486-bib-0037] Harley, E. , Fowler, K. , & Cotton, S. (2010). No detectable fertility benefit from a single additional mating in wild stalk‐eyed flies. PLoS One, 5(12), e14309 https://doi.org/10.1371/journal.pone.0014309 21179210 10.1371/journal.pone.0014309 PMC3001463

[ece33486-bib-0038] Harrison, X. A. (2014). Using observation‐level random effects to model overdispersion in count data in ecology and evolution. PeerJ, 2, e616 https://doi.org/10.7717/peerj.616 25320683 10.7717/peerj.616 PMC4194460

[ece33486-bib-0039] Harrison, X. A. (2015). A comparison of observation‐level random effect and Beta‐Binomial models for modelling overdispersion in Binomial data in ecology and evolution. PeerJ, 3, e1114 https://doi.org/10.7717/peerj.1114 26244118 10.7717/peerj.1114 PMC4517959

[ece33486-bib-0040] Hingle, A. , Fowler, K. , & Pomiankowski, A. (2001). Size‐dependent mate preference in the stalk‐eyed fly *Cyrtodiopsis dalmanni* . Animal Behaviour, 61(3), 589–595. https://doi.org/10.1006/anbe.2000.1613 10.1098/rspb.2001.1647 PMC1088732 11410149

[ece33486-bib-0041] Holman, L. , Price, T. A. R. , Wedell, N. , & Kokko, H. (2015). Coevolutionary dynamics of polyandry and sex‐linked meiotic drive. Evolution, 69(3), 709–720. https://doi.org/10.1111/evo.12595 25565579 10.1111/evo.12595

[ece33486-bib-0042] Hosken, D. J. , & Stockley, P. (2003). Benefits of polyandry: A life history perspective. Evolutionary Biology, 33, 173–194. https://doi.org/10.1007/978-1-4757-5190-1_4

[ece33486-bib-0043] House, C. M. , Walling, C. A. , Stamper, C. E. , & Moore, A. J. (2009). Females benefit from multiple mating but not multiple mates in the burying beetle *Nicrophorus vespilloides* . Journal of Evolutionary Biology, 22(9), 1961–1966. https://doi.org/10.1111/j.1420-9101.2009.01800.x 19682308 10.1111/j.1420-9101.2009.01800.x

[ece33486-bib-0044] Iwasa, Y. , & Pomiankowski, A. (1999). Good parent and good genes models of handicap evolution. Journal of Theoretical Biology, 200(1), 97–109. https://doi.org/10.1006/jtbi.1999.0979 10479542 10.1006/jtbi.1999.0979

[ece33486-bib-0045] Jennions, M. D. , & Petrie, M. (2000). Why do females mate multiply? A review of the genetic benefits. Biological Reviews, 75(1), 21–64. https://doi.org/10.1017/S0006323199005423 10740892 10.1017/s0006323199005423

[ece33486-bib-0046] Jones, T. M. (2001). A potential cost of monandry in the lekking sandfly *Lutzomyia Longipalpis* . Journal of Insect Behavior, 14(3), 385–399. https://doi.org/10.1023/A:1011127514317

[ece33486-bib-0047] Kelly, C. D. , & Jennions, M. D. (2011). Sexual selection and sperm quantity: Meta‐analyses of strategic ejaculation. Biological Reviews, 86(4), 863–884. https://doi.org/10.1111/j.1469-185X.2011.00175.x 21414127 10.1111/j.1469-185X.2011.00175.x

[ece33486-bib-0048] Kotiaho, J. S. , Simmons, L. W. , & Tomkins, J. L. (2001). Towards a resolution of the lek paradox. Nature, 410(6829), 684–686. https://doi.org/10.1038/35070557 11287953 10.1038/35070557

[ece33486-bib-0049] Kotrba, M. (1993) Das Reproduktionssystem von *Cyrtodiopsis whitei* Curran (Diopsidae, Diptera) unter besonderer Berücksichtigung der inneren weiblichen Geschlechtsorgane. Bonner Zoologischen Monographien, 33, 1–115.

[ece33486-bib-0050] Kotrba, M. (1995). The internal female genital organs of *Chaetodiopsis* and *Diasemopsis* (Diptera: Diopsidae) and their systematic relevance. Annals of the Natal Museum, 36(1989), 147–159.

[ece33486-bib-0051] Kotrba, M. (1996). Sperm transfer by spermatophore in Diptera: New results from the Diopsidae. Zoological Journal of the Linnean Society, 117, 305–323. https://doi.org/10.1111/j.1096-3642.1996.tb02192.x

[ece33486-bib-0052] Levin, E. , Mitra, C. , & Davidowitz, G. (2016). Fed males increase oviposition in female hawkmoths via non‐nutritive direct benefits. Animal Behaviour, 112, 111–118. https://doi.org/10.1016/j.anbehav.2015.11.019

[ece33486-bib-0053] Linklater, J. R. , Wertheim, B. , Wigby, S. , & Chapman, T. (2007). Ejaculate depletion patterns evolve in response to experimental manipulation of sex ratio in *Drosophila melanogaster* . Evolution, 61(8), 2027–2034. https://doi.org/10.1111/j.1558-5646.2007.00157.x 17683443 10.1111/j.1558-5646.2007.00157.x

[ece33486-bib-0054] Lorch, P. D. , Wilkinson, G. S. , & Reillo, P. R. (1993). Copulation duration and sperm precedence in the stalk‐eyed fly *Cyrtodiopsis whitei* (Diptera : Diopsidae). Behavioral Ecology and Sociobiology, 32(5), 303–311. https://doi.org/10.1007/BF00183785

[ece33486-bib-0055] Mautz, B. S. , Møller, A. P. , & Jennions, M. D. (2013). Do male secondary sexual characters signal ejaculate quality? A meta‐analysis. Biological Reviews, 88(3), 669–682. https://doi.org/10.1111/brv.12022 23374138 10.1111/brv.12022

[ece33486-bib-0056] Navara, K. J. , Anderson, E. M. , & Edwards, M. L. (2012). Comb size and color relate to sperm quality: A test of the phenotype‐linked fertility hypothesis. Behavioral Ecology, 23(5), 1036–1041. https://doi.org/10.1093/beheco/ars068

[ece33486-bib-0057] O'Dea, R. E. , Jennions, M. D. , & Head, M. L. (2014). Male body size and condition affects sperm number and production rates in mosquitofish, *Gambusia holbrooki* . Journal of Evolutionary Biology, 27(12), 2739–2744. https://doi.org/10.1111/jeb.12534 25403851 10.1111/jeb.12534

[ece33486-bib-0058] Orr, T. J. , & Brennan, P. L. R. (2015). Sperm storage: Distinguishing selective processes and evaluating criteria. Trends in Ecology & Evolution, 30(5), 261–272. https://doi.org/10.1016/j.tree.2015.03.006 25843274 10.1016/j.tree.2015.03.006

[ece33486-bib-0059] Parker, G. A. (1982). Why are there so many tiny sperm? Sperm competition and the maintenance of two sexes. Journal of Theoretical Biology, 96(2), 281–294. https://doi.org/10.1016/0022-5193(82)90225-9 7121030 10.1016/0022-5193(82)90225-9

[ece33486-bib-0060] Perez‐Staples, D. , Aluja, M. , Macías‐Ordóñez, R. , & Sivinski, J. (2008). Reproductive trade‐offs from mating with a successful male: The case of the tephritid fly *Anastrepha obliqua* . Behavioral Ecology and Sociobiology, 62(8), 1333–1340. https://doi.org/10.1007/s00265-008-0561-0

[ece33486-bib-0061] Perry, J. C. , & Rowe, L. (2010). Condition‐dependent ejaculate size and composition in a ladybird beetle. Proceedings of the Royal Society B: Biological Sciences, 277(1700), 3639–3647. https://doi.org/10.1098/rspb.2010.0810 20573622 10.1098/rspb.2010.0810 PMC2982242

[ece33486-bib-0062] Perry, J. C. , Sirot, L. K. , & Wigby, S. (2013). The seminal symphony: How to compose an ejaculate. Trends in Ecology & Evolution,28(7), 414–422. https://doi.org/10.1016/j.tree.2013.03.005 23582755 10.1016/j.tree.2013.03.005 PMC4974483

[ece33486-bib-0063] Pitcher, T. E. , Neff, B. D. , Rodd, F. H. , & Rowe, L. (2003). Multiple mating and sequential mate choice in guppies: Females trade up. Proceedings of the Royal Society B: Biological Sciences, 270(1524), 1623–1629. https://doi.org/10.1098/rspb.2002.2280 12908984 10.1098/rspb.2002.2280 PMC1691420

[ece33486-bib-0064] Pitnick, S. , Markow, T. A. , & Spicer, G. S. (1999). Evolution of multiple kinds of female sperm‐storage organs in *Drosophila* . Evolution, 53(6), 1804–1822. https://doi.org/10.2307/2640442 28565462 10.1111/j.1558-5646.1999.tb04564.x

[ece33486-bib-0065] Pizzari, T. , Jensen, P. , & Cornwallis, C. K. (2004). A novel test of the phenotype‐linked fertility hypothesis reveals independent components of fertility. Proceedings of the Royal Society B: Biological Sciences, 271(1534), 51–58. https://doi.org/10.1098/rspb.2003.2577 15002771 10.1098/rspb.2003.2577 PMC1691554

[ece33486-bib-0066] Presgraves, D. C. , Severance, E. , & Wilkinson, G. S. (1997). Sex chromosome meiotic drive in stalk‐eyed flies. Genetics, 147(3), 1169–1180. 9383060 10.1093/genetics/147.3.1169 PMC1208241

[ece33486-bib-0067] R Core Team (2016). R: A language and environment for statistical computing. Vienna, Austria: R Foundation for Statistical Computing Retrieved from https://www.r-project.org/

[ece33486-bib-0068] Ridley, M. (1988). Mating frequency and fecundity in insects. Biological Reviews, 63(4), 509–549. https://doi.org/10.1111/j.1469-185X.1988.tb00669.x

[ece33486-bib-0069] Rogers, D. W. , Chapman, T. , Fowler, K. , & Pomiankowski, A. (2005). Mating‐induced reduction in accessory reproductive organ size in the stalk‐eyed fly *Cyrtodiopsis dalmanni* . BMC Evolutionary Biology, 5, 37 https://doi.org/10.1186/1471-2148-5-37 15946387 10.1186/1471-2148-5-37 PMC1180822

[ece33486-bib-0070] Rogers, D. W. , Denniff, M. , Chapman, T. , Fowler, K. , & Pomiankowski, A. (2008). Male sexual ornament size is positively associated with reproductive morphology and enhanced fertility in the stalk‐eyed fly *Teleopsis dalmanni* . BMC Evolutionary Biology, 8, 236 https://doi.org/10.1186/1471-2148-8-236 18710553 10.1186/1471-2148-8-236 PMC2562384

[ece33486-bib-0071] Rogers, D. W. , Grant, C. A. , Chapman, T. , Pomiankowski, A. , & Fowler, K. (2006). The influence of male and female eyespan on fertility in the stalk‐eyed fly, *Cyrtodiopsis dalmanni* . Animal Behaviour, 72(6), 1363–1369. https://doi.org/10.1016/j.anbehav.2006.03.027

[ece33486-bib-0072] Rose, E. G. , Brand, C. L. , & Wilkinson, G. S. (2014). Rapid evolution of asymmetric reproductive incompatibilities in stalk‐eyed flies. Evolution, 68(2), 384–396. https://doi.org/10.1111/evo.12307 24171729 10.1111/evo.12307

[ece33486-bib-0073] Schneider, C. A. , Rasband, W. S. , & Eliceiri, K. W. (2012). NIH Image to ImageJ: 25 years of image analysis. Nature Methods, 9(7), 671–675. https://doi.org/10.1038/nmeth.2089 22930834 10.1038/nmeth.2089 PMC5554542

[ece33486-bib-0074] Sheldon, B. C. (1994). Male phenotype, fertility, and the pursuit of extra‐pair copulations by female birds. Proceedings of the Royal Society B: Biological Sciences, 257(1348), 25–30. https://doi.org/10.1098/rspb.1994.0089

[ece33486-bib-0075] Skaug, H. J. , Fournier, D. A. , Bolker, B. , Magnusson, A. , & Nielsen, A. (2016). Generalized linear mixed models using “AD Model Builder”. Retrieved from http://glmmadmb.r-forge.r-project.org

[ece33486-bib-0076] Slatyer, R. A. , Mautz, B. S. , Backwell, P. R. Y. , & Jennions, M. D. (2012). Estimating genetic benefits of polyandry from experimental studies: A meta‐analysis. Biological Reviews, 87(1), 1–33. https://doi.org/10.1111/j.1469-185X.2011.00182.x 21545390 10.1111/j.1469-185X.2011.00182.x

[ece33486-bib-0077] Small, J. , Cotton, S. , Fowler, K. , & Pomiankowski, A. (2009). Male eyespan and resource ownership affect contest outcome in the stalk‐eyed fly, *Teleopsis dalmanni* . Animal Behaviour, 78(5), 1213–1220. https://doi.org/10.1016/j.anbehav.2009.08.009

[ece33486-bib-0078] South, S. H. , & Arnqvist, G. (2008). Evidence of monandry in a mosquito (*Sabethes cyaneus*) with elaborate ornaments in both sexes. Journal of Insect Behavior, 21(6), 451–459. https://doi.org/10.1007/s10905-008-9137-0

[ece33486-bib-0079] Stewart, A. D. , Morrow, E. H. , & Rice, W. R. (2005). Assessing putative interlocus sexual conflict in *Drosophila melanogaster* using experimental evolution. Proceedings of the Royal Society B: Biological Sciences, 272(1576), 2029–2035. https://doi.org/10.1098/rspb.2005.3182 16191613 10.1098/rspb.2005.3182 PMC1559894

[ece33486-bib-0080] Tazzyman, S. J. , Pizzari, T. , Seymour, R. M. , & Pomiankowski, A. (2009). The evolution of continuous variation in ejaculate expenditure strategy. The American Naturalist, 174(3), E71–E82. https://doi.org/10.1086/603612 10.1086/603612 19627229

[ece33486-bib-0081] Toft, S. , & Albo, M. J. (2015). Optimal numbers of matings: The conditional balance between benefits and costs of mating for females of a nuptial gift‐giving spider. Journal of Evolutionary Biology, 28(2), 457–467. https://doi.org/10.1111/jeb.12581 25580948 10.1111/jeb.12581

[ece33486-bib-0082] Torres‐Vila, L. M. , & Jennions, M. D. (2005). Male mating history and female fecundity in the Lepidoptera: Do male virgins make better partners? Behavioral Ecology and Sociobiology, 57(4), 318–326. https://doi.org/10.1007/s00265-004-0857-7

[ece33486-bib-0083] Tregenza, T. , & Wedell, N. (2002). Polyandrous females avoid costs of inbreeding. Nature, 415(6867), 71–73. https://doi.org/10.1038/415071a 11780118 10.1038/415071a

[ece33486-bib-0084] Tuni, C. , Albo, M. J. , & Bilde, T. (2013). Polyandrous females acquire indirect benefits in a nuptial feeding species. Journal of Evolutionary Biology, 26(6), 1307–1316. https://doi.org/10.1111/jeb.12137 23639113 10.1111/jeb.12137

[ece33486-bib-0085] Wang, Q. , & Davis, L. K. (2006). Females remate for sperm replenishment in a seed bug: Evidence from offspring viability. Journal of Insect Behavior, 19(3), 337–346. https://doi.org/10.1007/s10905-006-9027-2

[ece33486-bib-0086] Warner, R. R. , Shapiro, D. Y. , Marcanato, A. , & Petersen, C. W. (1995). Sexual conflict: Males with highest mating success convey the lowest fertilization benefits to females. Proceedings of the Royal Society B: Biological Sciences, 262(1364), 135–139. https://doi.org/10.1098/rspb.1995.0187 8524908 10.1098/rspb.1995.0187

[ece33486-bib-0087] Wedell, N. , Gage, M. J. G. , & Parker, G. A. (2002). Sperm competition, male prudence and sperm‐limited females. Trends in Ecology & Evolution, 17(7), 313–320. https://doi.org/10.1016/S0169-5347(02)02533-8

[ece33486-bib-0088] Wedell, N. , & Ritchie, M. G. (2004). Male age, mating status and nuptial gift quality in a bushcricket. Animal Behaviour, 67(6), 1059–1065. https://doi.org/10.1016/j.anbehav.2003.10.007

[ece33486-bib-0089] Wilgers, D. J. , & Hebets, E. A. (2012). Age‐related female mating decisions are condition dependent in wolf spiders. Behavioral Ecology and Sociobiology, 66(1), 29–38. https://doi.org/10.1007/s00265-011-1248-5

[ece33486-bib-0090] Wilkinson, G. S. , Amitin, E. G. , & Johns, P. M. (2005). Sex‐linked correlated responses in female reproductive traits to selection on male eye span in stalk‐eyed flies. Integrative and Comparative Biology, 45(3), 500–510. https://doi.org/10.1093/icb/45.3.500 21676795 10.1093/icb/45.3.500

[ece33486-bib-0091] Wilkinson, G. S. , Johns, P. M. , Kelleher, E. S. , Muscedere, M. L. , & Lorsong, A. (2006). Fitness effects of X chromosome drive in the stalk‐eyed fly, *Cyrtodiopsis dalmanni* . Journal of Evolutionary Biology, 19(6), 1851–1860. https://doi.org/10.1111/j.1420-9101.2006.01169.x 17040382 10.1111/j.1420-9101.2006.01169.x

[ece33486-bib-0092] Wilkinson, G. S. , Kahler, H. , & Baker, R. H. (1998). Evolution of female mating preferences in stalk‐eyed flies. Behavioral Ecol ogy, 9(5), 525–533. https://doi.org/10.1093/beheco/9.5.525

[ece33486-bib-0093] Wilkinson, G. S. , Swallow, J. G. , Christianson, S. J. , & Madden, K. (2003). Phylogeography of sex ratio and multiple mating in stalk‐eyed flies from southeast Asia. Genetica, 117, 37–46. 12656571 10.1023/a:1022360531703

[ece33486-bib-0094] Wright, L. I. , Fuller, W. J. , Godley, B. J. , McGowan, A. , Tregenza, T. , & Broderick, A. C. (2013). No benefits of polyandry to female green turtles. Behavioral Ecology, 24(4), 1022–1029. https://doi.org/10.1093/beheco/art003

[ece33486-bib-0095] Yasui, Y. (1998). The “genetic benefits” of female multiple mating reconsidered. Trends in Ecology & Evolution, 13(6), 246–250. https://doi.org/10.1016/S0169-5347(98)01383-4 21238286 10.1016/s0169-5347(98)01383-4

[ece33486-bib-0096] Zeh, J. A. , & Zeh, D. W. (1996). The evolution of polyandry I: Intragenomic conflict and genetic incompatibility. Proceedings of the Royal Society B: Biological Sciences, 263(1377), 1711–1717. https://doi.org/10.1098/rspb.1996.0250

[ece33486-bib-0097] Zeh, J. A. , & Zeh, D. W. (2001). Reproductive mode and the genetic benefits of polyandry. Animal Behaviour, 61(6), 1051–1063. https://doi.org/10.1006/anbe.2000.1705

