# Supplementary material for: Variation in the benefits of multiple mating on female fertility in wild stalk‐eyed flies
Source: Ecol Evol. 2017 Oct 24;7(23):10103–15. doi: 10.1002/ece3.3486 (PMC5723596; doi:10.1002/ece3.3486)
Supplement: Supplementary file 1 [file ECE3-7-10103-s001.pdf]

Variation in the benefits of multiple mating on female fertility in wild stalk-eyed flies.

Supporting Information

Lara Meade
Elisabeth Harley
Alison Cotton
James M. Howie
Andrew Pomiankowski
Kevin Fowler

Contents

Overview **2**

Experiment 1: Gains from an additional mating **3**
A) Variation in fecundity 3
B) Variation in fertility 8

Experiment 2: Investigation of female and male effects **20**
C) Variation in fecundity 20
D) Variation in fertility 25

Overview

We present tables and effect size estimates for GLMM models summarised in the main text. The tables are split into two sections, corresponding to the pair of experiments described in the main text, "*Gains from an additional mating*" and "*Investigation of female and male effects*".

For each of fecundity, fertility and proportion fertility, we compared patterns within and between the periods before and after mating. The models refer to these periods as "Groups". In some models, Groups are the whole periods before and after mating (B/A), each consisting of four counts over eight days. Additionally, in other models, Groups refer the count immediately prior to and immediately after mating, each consisting of two days (days 11-12 and 14-15 denoted 12/15, experiment 1; or days 11-12 and 13-14 denoted 12/14, experiment 2). Models all have an observation-level random effect (OLRE) and, where relevant, stream site and female ID are also random effects. Random effects are denoted (1|effect) in models. For each analysis, the full model is reported together with a table of Chi-squared values and P-values from model comparisons after removal of the variable of interest. This is followed by a table of coefficients of the fixed effects, and then a table of the variance between levels of the random effects. For each experiment, the initial analysis examining patterns in fecundity, fertility or proportion fertility over time prior to mating also reports on model comparisons with removal of random effects. In addition, experiment 2 involves a pair of male types; sperm depleted (SD) or non-sperm depleted (NSD).

Experiment 1: Gains from an additional mating

A) Variation in fecundity

A.S1) Variation in fecundity over time prior to an additional mating

```
model1 = glmer(Fecundity ~ Day + (1|Stream) + (1|Female.ID) + (1|OLRE), data = Before,
               family = poisson)
```

Model comparison:

	ChiSq	P
Stream	2.8652	0.09051
Female ID	5.3291	0.02097
Day	1.1815	0.277

Fixed effects:

```
             Estimate Std.Error z.value      P
(Intercept)  0.84950   0.61180   1.389 0.1650
Day         -0.06627   0.06132  -1.081 0.2798
```

Random effects:

```
          Variance
Female.ID   0.6073
Stream      0.4624
```

Sample size:

```
Observations    Female.ID       Stream
         144           36           11
```

A.S2) Variation in fecundity over time after an additional mating

```
model1 = glmer(Fecundity ~ Day + (1|Stream) + (1|Female.ID) + (1|OLRE), data = After,
               family = poisson)
```

Model comparison:

	ChiSq	P
Stream	0.0676	0.7948
Female ID	24.5018	7.424×10^{-7}
Day	4.9927	0.02545

Fixed effects:

```
            Estimate Std.Error z.value       P
(Intercept)   2.5340   1.06000   2.392 0.01678
Day          -0.1291   0.05722  -2.257 0.02402
```

Random effects:

```
          Variance
Female.ID   1.9174
Stream      0.1141
```

Sample size:

```
Observations    Female.ID       Stream
         144           36           11
```

A.S3) Variation in fecundity across all days

```
model1 = glmer(Fecundity ~ Day + (1|Stream) + (1|Female.ID) + (1|OLRE), data = All,
               family = poisson)
```

Model comparison:

	ChiSq	P
Day	1.2586	0.2619

Fixed effects:

```
            Estimate Std.Error z.value          P
(Intercept)   0.5232 0.0007437  703.60  0.000e+00
Day          -0.0218 0.0007472  -29.18 3.729e-187
```

Random effects:

```
          Variance
Female.ID   1.2165
Stream      0.2680
```

Sample size:

```
Observations    Female.ID       Stream
         288           36           11
```

A.S4) Total individual fecundity before and after an additional mating

```
model1 = glmer(TotFecundity ~ Group + (1|Stream) + (1|Female.ID) + (1|OLRE), data = Total,
               family = poisson)
```

Model comparison:

	ChiSq	P
Group (B/A)	0.1001	0.7517

Fixed effects:

```
             Estimate Std.Error z.value         P
(Intercept)   2.21300    0.2705  8.1810 2.809e-16
Group2.After -0.05855    0.1845 -0.3173 7.510e-01
```

Random effects:

```
          Variance
Female.ID   0.8604
Stream      0.3275
```

Sample size:

```
Observations    Female.ID       Stream
          72           36           11
```

A.S5) Total individual fecundity between day 12 and day 15

```
model1 = glmer(Fecundity ~ Group + (1|Stream) + (1|Female.ID) + (1|OLRE), data = Day12v15,
               family = poisson)
```

Model comparison:

	ChiSq	P
Group (12/15)	2.4907	0.1145

Fixed effects:

```
             Estimate Std.Error z.value      P
(Intercept)    0.1171    0.4740   0.247 0.8049
GroupDay15     0.3696    0.2298   1.608 0.1078
```

Random effects:

```
          Variance
Female.ID   4.2621
Stream      0.2425
```

Sample size:

```
Observations    Female.ID       Stream
          72           36           11
```

B) Variation in fertility

B.S1) Variation in total fertility with total fecundity prior to an additional mating

```
model1 = glmer(TotFertility ~ TotFecundity + (1|Stream) + (1|OLRE), data = TotalBefore,
               family = poisson)
```

Model comparison:

	ChiSq	P
TotFecundity	5.9894	0.01439

Fixed effects:

```
             Estimate Std.Error z.value       P
(Intercept)  -0.10130   0.50230 -0.2016 0.84020
TotFecundity  0.03429   0.01489  2.3030 0.02126
```

Random effects:

```
       Variance
Stream   0.7826
```

Sample size:

```
Observations       Stream
          36           11
```

B.S2) Variation in total fertility with total fecundity after an additional mating

```
model1 = glmer(TotFertility ~ TotFecundity + (1|Stream) + (1|OLRE), TotalAfter,
               family = poisson)
```

Model comparison:

	ChiSq	P
TotFecundity	22.6367	1.957×10^{-6}

Fixed effects:

```
             Estimate Std.Error z.value         P
(Intercept)   0.31830  0.390100   0.816 4.145e-01
TotFecundity  0.05308  0.009147   5.803 6.497e-09
```

Random effects:

```
       Variance
Stream    0.453
```

Sample size:

```
Observations       Stream
          32           11
```

B.S3) Variation in fertility over time prior to an additional mating

```
model1 = glmer(Fertility ~ Day + (1|Stream) + (1|Female.ID) + (1|OLRE), data = Before,
               family = poisson)
```

Model comparison:

	Chi Sq	P
Stream	5.8958	0.01518
Female ID	5.7493	0.0165
Day	8.4502	0.00365

Fixed effects:

```
            Estimate Std.Error z.value        P
(Intercept)    1.164   0.57970   2.008 0.044670
Day           -0.168   0.05626  -2.986 0.002828
```

Random effects:

```
          Variance
Female.ID   0.5756
Stream      0.7800
```

Sample size:

```
Observations    Female.ID      Stream
          84           36          11
```

B.S4) Variation in proportion fertility over time prior to an additional mating

```
model1 = glmer(Prop.Fertility ~ Day  + (1|Stream) + (1|Female.ID) + (1|OLRE),
               data = Before, family = binomial)
```

Model comparison:

	ChiSq	P
Stream	4.3233	0.03759
Female ID	20.5766	5.729×10^{-6}
Day	17.5402	2.813×10^{-5}

Fixed effects:

```
            Estimate Std.Error z.value         P
(Intercept)   0.9179   0.78510   1.169 2.424e-01
Day          -0.2789   0.06416  -4.347 1.378e-05
```

Random effects:

```
          Variance
Female.ID   2.2974
Stream      2.2500
```

Sample size:

```
Observations    Female.ID       Stream
          84           36           11
```

B.S5) Variation in fertility over time after an additional mating

```
model1 = glmer(Fertility ~ Day + (1|Stream) + (1|Female.ID) + (1|OLRE), data = After,
               family = poisson)
```

Model comparison:

	Chi Sq	P
Stream	1.4439	0.2295
Female ID	9.7932	0.001752
Day	2.1722	0.1405

Fixed effects:

```
             Estimate Std.Error z.value      P
(Intercept)   2.0720   1.30100   1.593 0.1112
Day          -0.1043   0.07077  -1.474 0.1405
```

Random effects:

```
          Variance
Female.ID   1.3584
Stream      0.6006
```

Sample size:

```
Observations    Female.ID       Stream
          85           32           11
```

B.S6) Variation in proportion fertility over time after an additional mating

```
model1 = glmer(Prop.Fertility ~ Day + (1|Stream) + (1|Female.ID) + (1|OLRE), data = After,
               family = binomial)
```

Model comparison:

	ChiSq	P
Stream	5.5951	0.01801
Female ID	3.4542	0.06309
Day	0.5063	0.4767

Fixed effects:

```
            Estimate Std.Error z.value      P
(Intercept)    1.742    2.9330  0.5941 0.5525
Day           -0.114    0.1613 -0.7068 0.4797
```

Random effects:

```
          Variance
Female.ID   2.5136
Stream      4.0503
```

Sample size:

```
Observations    Female.ID      Stream
          85           32          11
```

B.S7) Total individual fertility before and after an additional mating

```
model1 = glmer(TotFertility ~ Group + (1|Stream) + (1|Female.ID) + (1|OLRE), data = Total,
               family = poisson)
```

Model comparison:

	ChiSq	P
Group (B/A)	3.5892	0.05816

Fixed effects:

```
             Estimate Std.Error z.value       P
(Intercept)    0.3536    0.4327  0.8172 0.41380
Group2.After   0.8616    0.4509  1.9110 0.05605
```

Random effects:

```
          Variance
Female.ID    0.000
Stream       0.751
```

Sample size:

```
Observations    Female.ID       Stream
          68           36           11
```

B.S8) Total proportion individual fertility before and after an additional mating

```
model1 = glmer(Prop.Fertility ~ Group + (1|Stream) + (1|Female.ID) + (1|OLRE),
                    data = Total, family = binomial)
```

Model comparison:

	ChiSq	P
Group	5.153	0.02321

Fixed effects:

```
             Estimate Std.Error z.value       P
(Intercept)    -1.469    0.4929  -2.979 0.00289
Group2.After    1.307    0.5595   2.336 0.01948
```

Random effects:

```
          Variance
Female.ID   0.0000
Stream      0.8011
```

Sample size:

```
Observations    Female.ID       Stream
          68           36           11
```

B.S9) Total individual fertility between day 12 and day 15

```
model1 = glmer(Fertility ~ Group + (1|Stream) + (1|Female.ID) + (1|OLRE), data = Day12v15,
               family = poisson)
```

Model comparison:

	ChiSq	P
Group (12/15)	10.0766	0.001502

Fixed effects:

```
             Estimate Std.Error z.value         P
(Intercept)   -0.6121    0.5317  -1.151 0.2496000
GroupDay15     1.6650    0.4513   3.689 0.0002251
```

Random effects:

```
          Variance
Female.ID   0.8276
Stream      0.5030
```

Sample size:

```
Observations    Female.ID       Stream
          41           24           10
```

B.S10) Total individual proportion fertility between day 12 and day 15

```
model1 = glmer(Prop.Fertility ~ Group + (1|Stream) + (1|Female.ID) + (1|OLRE),
               Day12v15, family = binomial)
```

Model comparison:

	ChiSq	P
Group (12/15)	15.5344	8.102×10^{-5}

Fixed effects:

```
            Estimate Std.Error z.value         P
(Intercept)   -3.207    0.9602  -3.340 8.378e-04
GroupDay15     3.431    0.8366   4.101 4.118e-05
```

Random effects:

```
          Variance
Female.ID   0.0000
Stream      3.6911
```

Sample size:

```
Observations    Female.ID       Stream
          41           24           10
```

B.S11) Direction of change in fertility before and after an additional mating

```
model1a = glmer(FertilityChange ~ FecundityBefore + (1|Stream), data = dataBA, family = binomial)
```

```
model1b = glmer(FertilityChange ~ FertilityBefore + (1|Stream), data = dataBA, family = binomial)
```

```
model1c = glmer(FertilityChange ~ FecundityBefore + FertilityBefore + (1|Stream),
            data = dataBA, family = binomial)
```

Model comparison:

	ChiSq	P
Fecundity	2.2001	0.138
Fertility	5.8261	0.01579
Relative Fecundity	18.3375	1.85×10^{-5}
Relative Fertility	21.9635	2.779×10^{-6}

Fixed effects:

```
                     Estimate Std.Error z.value       P
(Intercept 1a)        -0.7495     0.653  -1.148  0.2511
1a FecundityBefore    0.03305   0.02603    1.27  0.2042

(Intercept 1b)         0.5516    0.4736   1.165  0.2442
1b FertilityBefore    -0.1559   0.08398  -1.857 0.06335

(Intercept 1c)         -1.156    0.8439   -1.37  0.1708
1c FecundityBefore     0.3001    0.1328    2.26 0.02379
1c FertilityBefore     -1.258    0.6316  -1.992 0.04642
```

Random effects:

```
           Variance
1a Stream    0.5952
1b Stream    0.0000
1c Stream    0.0000
```

Sample size:

```
Observations       Stream
          32           11
```

B.S12) Degree of change in proportion fertility before and after an additional mating

```
model1a = lmer(PropChange ~  FecundityBefore + (1|Stream), data = dataBA)
```

```
model1b = lmer(PropChange ~  FertilityBefore + (1|Stream), data = dataBA)
```

```
model1c = lmer(PropChange ~  FecundityBefore + FertilityBefore + (1|Stream), data = dataBA)
```

Model comparison:

	ChiSq	P
Fecundity	7.5575	0.005976
Fertility	2.0648	0.1507
Relative Fecundity	12.842	3.389×10^{-4}
Relative Fertility	7.3493	0.006709

Fixed effects:

	Estimate	Std.Error	z.value	P	NA
(Intercept 1a)	-0.07952	0.1423	13.88	-0.559	0.5851
1a FecundityBefore	0.0123	0.003987	26.81	3.086	0.004667
(Intercept 1b)	0.2384	0.1156	8.521	2.061	0.07107
1b FertilityBefore	-0.01568	0.01122	14.15	-1.397	0.1838
(Intercept 1c)	0.02965	0.113	11.37	0.2623	0.7978
1c FecundityBefore	0.01567	0.004105	28.77	3.818	0.0006604
1c FertilityBefore	-0.03133	0.01049	19.21	-2.986	0.00753

Random effects:

	Variance
1a Stream	0.1157
1b Stream	0.0271
1c Stream	0.0284

Sample size:

Observations	Stream
32	11

Experiment 2: Investigation of female and male effects

C) Variation in fecundity

C.S1) Variation in fecundity over time prior to an additional mating

```
model1 = glmer(Fecundity ~ Day * MaleType + (1|Stream) + (1|Female.ID) + (1|OLRE),
              data = Before, family = poisson)
```

Model comparison:

	ChiSq	P
Female ID	23.4754	1.265×10^{-6}
MaleType (SD/NSD) x Day	0.0336	0.8546
MaleType (SD/NSD)	1.0766	0.2994
Day	33.7777	6.178×10^{-9}

Fixed effects:

```
               Estimate Std.Error z.value        P
(Intercept)    -1.89600   0.73010 -2.5970 0.009412
Day             0.26640   0.06956  3.8300 0.000128
MaleTypeSD      0.24610   0.95140  0.2587 0.795800
Day:MaleTypeSD  0.01678   0.09155  0.1833 0.854600
```

Random effects:

```
          Variance
Female.ID   0.9057
```

Sample size:

```
Observations    Female.ID       Stream
         136           34            5
```

C.S2) Variation in fecundity over time and between male types (sperm depleted / non-sperm depleted) after an additional mating

```
model1 = glmer(Fecundity ~ Day * MaleType + (1|Stream) + (1|Female.ID) + (1|OLRE),
             data = After, family = poisson)
```

Model comparison:

	ChiSq	P
Female ID	12.3373	4.44×10^{-4}
MaleType (SD/NSD) x Day	0.85	0.3566
MaleType (SD/NSD)	0.3047	0.581
Day	37.8321	7.71×10^{-10}

Fixed effects:

```
               Estimate Std.Error z.value         P
(Intercept)     5.39300   1.29300  4.1690 0.0000306
Day            -0.27120   0.07633 -3.5530 0.0003804
MaleTypeSD      1.75200   1.72600  1.0150 0.3100000
Day:MaleTypeSD -0.09379   0.10180 -0.9214 0.3568000
```

Random effects:

```
          Variance
Female.ID   0.6181
```

Sample size:

```
Observations    Female.ID       Stream
         136           34            5
```

C.S3) Variation in fecundity across all days

```
model1 = glmer(Fecundity ~ Day + (1|Stream) + (1|Female.ID) + (1|OLRE), data = All,
               family = poisson)
```

Model comparison:

	ChiSq	P
Day	0.2665	0.6057

Fixed effects:

```
             Estimate Std.Error z.value         P
(Intercept)  0.67970 0.0007596  894.90 0.000e+00
Day          0.01072 0.0007613   14.09 4.572e-45
```

Random effects:

```
          Variance
Female.ID   0.4259
```

Sample size:

```
Observations    Female.ID       Stream
         272           34            5
```

C.S4) Total individual fecundity before and after an additional mating and between male types

```
model1 = glmer(TotFecundity ~ Group * MaleType  + (1|Stream) + (1|Female.ID) + (1|OLRE),
            data = Total, family = poisson)
```

Model comparison:

	ChiSq	P
MaleType (SD/NSD) x Group (B/A)	0.4838	0.4867
MaleType (SD/NSD)	0.96	0.3272
Group (B/A)	2.0814	0.1491

Fixed effects:

```
                        Estimate Std.Error z.value         P
(Intercept)               2.3240    0.2643   8.790 1.497e-18
Group2.After              0.4160    0.2797   1.487 1.370e-01
MaleTypeSD                0.4210    0.3489   1.207 2.275e-01
Group2.After:MaleTypeSD  -0.2582    0.3704  -0.697 4.858e-01
```

Random effects:

```
          Variance
Female.ID   0.4262
```

Sample size:

```
Observations    Female.ID       Stream
          68           34            5
```

C.S5) Total individual fecundity between day 12 and day 14 and between male types

```
model1 = glmer(Fecundity ~ Group * MaleType + (1|Stream) + (1|Female.ID) + (1|OLRE),
              data = Day12v14, family = poisson)
```

Model comparison:

	ChiSq	P
MaleType (SD/NSD) x Group (12/14)	0.5267	0.468
MaleType (SD/NSD)	0.3591	0.549
Group (12/14)	2.7386	0.09795

Fixed effects:

```
                      Estimate Std.Error z.value         P
(Intercept)           1.606000    0.2904 5.53100 3.189e-08
GroupDay14            0.201300    0.3497 0.57560 5.649e-01
MaleTypeSD            0.005779    0.3861 0.01497 9.881e-01
GroupDay14:MaleTypeSD 0.340500    0.4646 0.73290 4.636e-01
```

Random effects:

```
          Variance
Female.ID   0.3151
```

Sample size:

```
Observations    Female.ID       Stream
          68           34            5
```

D) Variation in fertility

D.S1) Variation in total fertility with total fecundity prior to an additional mating

```
model1 = glmer(TotFertility ~ TotFecundity + (1|Stream) + (1|OLRE), data = TotalBefore,
               family = poisson)
```

Model comparison:

	ChiSq	P
TotFecundity	36.4477	1.568×10^{-9}

Fixed effects:

```
             Estimate Std.Error z.value         P
(Intercept)   0.60720  0.180500   3.364 7.692e-04
TotFecundity  0.03741  0.004727   7.915 2.467e-15
```

Sample size:

```
Observations       Stream
          33            5
```

D.S2) Variation in total fertility with total fecundity after an additional mating

```
model1 = glmer(TotFertility ~ TotFecundity + (1|Stream) + (1|OLRE) , TotalAfter,
              family = poisson)
```

Model comparison:

	ChiSq	P
TotFecundity	40.8785	1.62×10^{-10}

Fixed effects:

```
             Estimate Std.Error z.value         P
(Intercept)   1.64400  0.105900  15.510 2.758e-54
TotFecundity  0.01987  0.002248   8.838 9.779e-19
```

Sample size:

```
Observations       Stream
          33            5
```

D.S3) Variation in fertility over time prior to an additional mating

```
model1 = glmer(Fertility ~ Day + (1|Stream) + (1|Female.ID) + (1|OLRE), data = Before,
               family = poisson)
```

Model comparison:

	ChiSq	P
Female ID	4.7501	0.0293
Day	0.2539	0.6144

Fixed effects:

```
             Estimate Std.Error  z.value      P
(Intercept) -0.04793   0.56520 -0.08481 0.9324
Day          0.02807   0.05572  0.50380 0.6144
```

Random effects:

```
          Variance
Female.ID    0.378
```

Sample size:

```
Observations    Female.ID       Stream
          95           33            5
```

D.S4) Variation in proportion fertility over time prior to an additional mating

```
model1 = glmer(Prop.Fertility ~ Day + (1|Stream) + (1|Female.ID) + (1|OLRE), data = Before,
               family = binomial)
```

Model comparison:

	ChiSq	P
Female ID	4.0869	0.04322
Day	7.9025	0.004937

Fixed effects:

```
            Estimate Std.Error z.value        P
(Intercept)   0.5122   0.51120   1.002 0.316300
Day          -0.1502   0.04971  -3.022 0.002515
```

Random effects:

```
          Variance
Female.ID   0.2882
```

Sample size:

```
Observations    Female.ID       Stream
          95           33            5
```

D.S5) Variation in fertility over time and between male types after an additional mating

```
model1 = glmer(Fertility ~ Day * MaleType + (1|Stream) + (1|Female.ID) + (1|OLRE),
               data = After, family = poisson)
```

Model comparison:

	ChiSq	P
Female ID	0.327	0.5674
MaleType (SD/NSD) x Day	0.0201	0.8872
MaleType (SD/NSD)	0.1299	0.7186
Day	33.2647	8.043×10^{-9}

Fixed effects:

```
               Estimate Std.Error  z.value         P
(Intercept)      5.7590   1.15400  4.99200 5.979e-07
Day             -0.3067   0.07170 -4.27800 1.885e-05
MaleTypeSD      -0.1442   1.56500 -0.09211 9.266e-01
Day:MaleTypeSD   0.0139   0.09809  0.14170 8.873e-01
```

Random effects:

```
          Variance
Female.ID   0.0424
```

Sample size:

```
Observations    Female.ID       Stream
          97           33            5
```

D.S6) Variation in proportion fertility over time and between male types after an additional mating

```
model1 = glmer(Prop.Fertility ~ Day * MaleType + (1|Stream) + (1|Female.ID) + (1|OLRE),
            data = After, family = binomial)
```

Model comparison:

	ChiSq	P
Female ID	0.327	0.5674
MaleType (SD/NSD) x Day	0.1995	0.6552
MaleType (SD/NSD)	0.9201	0.3374
Day	31.2344	2.287×10^{-8}

Fixed effects:

```
               Estimate Std.Error z.value         P
(Intercept)     3.62100   1.16000  3.1220 0.0017980
Day            -0.24750   0.07210 -3.4320 0.0005981
MaleTypeSD      0.47910   1.50100  0.3191 0.7496000
Day:MaleTypeSD -0.04202   0.09388 -0.4476 0.6544000
```

Random effects:

```
          Variance
Female.ID        0
```

Sample size:

```
Observations    Female.ID       Stream
          97           33            5
```

D.S7) Total individual fertility before and after an additional mating and between male types

```
model1 = glmer(TotFertility ~ Group * MaleType + (1|Stream) + (1|Female.ID) + (1|OLRE),
            data = Total, family = poisson)
```

Model comparison:

	ChiSq	P
MaleType (SD/NSD) x Group (B/A)	0.6327	0.4264
MaleType (SD/NSD)	1.0901	0.2964
Group (B/A)	12.5805	3.898×10^{-4}

Fixed effects:

```
                        Estimate Std.Error z.value         P
(Intercept)               1.2030    0.2565  4.6890 2.744e-06
Group2.After              0.7751    0.2534  3.0590 2.223e-03
MaleTypeSD                0.4059    0.3045  1.3330 1.826e-01
Group2.After:MaleTypeSD  -0.2669    0.3293 -0.8106 4.176e-01
```

Random effects:

```
          Variance
Female.ID    0.222
```

Sample size:

```
Observations    Female.ID       Stream
          66           34            5
```

D.S8) Total proportion individual fertility before and after an additional mating and between male types

```
model1 = glmer(Prop.Fertility ~ Group * MaleType + (1|Female.ID) + (1|OLRE) + (1|Stream),
               data = Total, family = binomial)
```

Model comparison:

	ChiSq	P
MaleType (SD/NSD) x Group (B/A)	2.6744	0.102
MaleType (SD/NSD)	0.006	0.9384
Group (B/A)	12.4228	4.241×10^{-4}

Fixed effects:

```
                        Estimate Std.Error z.value         P
(Intercept)              -1.1520    0.2051  -5.616 1.957e-08
Group2.After              0.9001    0.2453   3.670 2.430e-04
MaleTypeSD                0.3224    0.2600   1.240 2.151e-01
Group2.After:MaleTypeSD  -0.5289    0.3125  -1.693 9.054e-02
```

Random effects:

```
          Variance
Female.ID   0.0817
```

Sample size:

```
Observations    Female.ID       Stream
          66           34            5
```

D.S9) Total individual fertility between day 12 and day 14 and between male types

```
model1 = glmer(Fertility ~ Group * MaleType + (1|Stream) + (1|Female.ID) + (1|OLRE),
              data = Day12v14, family = poisson)
```

Model comparison:

	ChiSq	P
MaleType (SD/NSD) x Group (12/14)	0.0027	0.9589
MaleType (SD/NSD)	0.3949	0.5297
Group (12/14)	23.8148	1.061×10^{-6}

Fixed effects:

```
                       Estimate Std.Error  z.value         P
(Intercept)              0.2518    0.3090  0.81500 0.4150000
GroupDay14               1.2330    0.3423  3.60300 0.0003143
MaleTypeSD               0.1831    0.3901  0.46940 0.6388000
GroupDay14:MaleTypeSD   -0.0227    0.4409 -0.05148 0.9589000
```

Random effects:

```
          Variance
Female.ID   0.2145
```

Sample size:

```
Observations    Female.ID       Stream
          62           34            5
```

D.S10) Total individual proprtion fertility between day 12 and day 14 and between male types

```
model1 = glmer(Prop.Fertility ~ Group * MaleType + (1|Stream) + (1|Female.ID) + (1|OLRE),
              data = Day12v14, family = binomial)
```

Model comparison:

	ChiSq	P
MaleType (SD/NSD) x Group (12/14)	0.2317	0.6303
MaleType (SD/NSD)	0.1717	0.6786
Group (12/14)	27.0669	1.965×10^{-7}

Fixed effects:

```
                      Estimate Std.Error z.value         P
(Intercept)            -1.4090    0.2925 -4.8190 1.446e-06
GroupDay14              1.4260    0.3541  4.0280 5.634e-05
MaleTypeSD              0.2421    0.3805  0.6363 5.246e-01
GroupDay14:MaleTypeSD  -0.2180    0.4513 -0.4831 6.290e-01
```

Random effects:

```
          Variance
Female.ID   0.1289
```

Sample size:

```
Observations    Female.ID       Stream
          62           34            5
```

D.S11) Direction of change in fertility before and after an additional mating

```
model1a = glmer(FertilityChange ~ FecundityBefore + (1|Stream), data = dataBA, family = binomial)

model1b = glmer(FertilityChange ~ FertilityBefore + (1|Stream), data = dataBA, family = binomial)

model1c = glmer(FertilityChange ~ FecundityBefore + FertilityBefore + MaleType + (1|Stream),
              data = dataBA, family = binomial)
```

Model comparison:

	ChiSq	P
Fecundity	4.7193	0.02983
Fertility	8.2079	0.004171
Relative Fecundity	0.1939	0.6597
Relative Fertility	3.6824	0.05499
MaleType (SD/NSD)	0.2076	0.6487

Fixed effects:

```
                   Estimate Std.Error z.value       P
(Intercept 1a)        2.316     1.031   2.246 0.02471
1a FecundityBefore -0.04942   0.02754  -1.794 0.07278

(Intercept 1b)        3.175     1.681   1.889 0.05888
1b FertilityBefore  -0.2983    0.2087   -1.43  0.1528

(Intercept 1c)        3.609     2.021   1.786 0.07418
1c FecundityBefore   0.0316   0.06389  0.4945  0.6209
1c FertilityBefore   -0.444    0.3681  -1.206  0.2278
1c MaleTypeSD       -0.4878      1.09 -0.4475  0.6545
```

Random effects:

```
          Variance
1a Stream   0.3917
1b Stream   1.2593
1c Stream   1.5571
```

Sample size:

```
Observations       Stream
          32            5
```

D.S12) Degree of change in proportion fertility before and after an additional mating and between male types

```
model1a = lmer(PropChange ~  FertilityBefore + (1|Stream), data = dataBA)
```

```
model1b = lmer(PropChange ~  FertilityBefore + (1|Stream), data = dataBA)
```

```
model1c = lmer(PropChange ~  FecundityBefore + FertilityBefore + MaleType + (1|Stream), data = dataBA)
```

Model comparison:

	ChiSq	P
Fecundity	0.0476	0.8274
Fertility	3.1064	0.07798
Relative Fecundity	4.4386	0.03514
Relative Fertility	7.4975	0.006179
MaleType (SD/NSD)	0.4654	0.4951

Fixed effects:

```
                     Estimate Std.Error z.value       P      NA
(Intercept 1a)         0.1297   0.06412   11.95   2.023 0.06605
1a FecundityBefore -0.0005093  0.002123   29.92 -0.2399  0.8121

(Intercept 1b)         0.1733   0.05553   7.095   3.121 0.01654
1b FertilityBefore  -0.009276  0.005172   29.99  -1.794 0.08296

(Intercept 1c)         0.1423   0.06768    15.1   2.103 0.05268
1c FecundityBefore    0.00667  0.003239   27.57   2.059 0.04903
1c FertilityBefore   -0.02193  0.008353    27.2  -2.625 0.01405
1c MaleTypeSD         -0.0507   0.07915      28 -0.6405   0.527
```

Random effects:

```
          Variance
1a Stream   0.0013
1b Stream   0.0021
1c Stream   0.0003
```

Sample size:

```
Observations       Stream
          32            5
```
